# Vacancies on 2D transition metal dichalcogenides elicit ferroptotic cell death

**DOI:** 10.1038/s41467-020-17300-7

**Published:** 2020-07-13

**Authors:** Shujuan Xu, Huizhen Zheng, Ronglin Ma, Di Wu, Yanxia Pan, Chunyang Yin, Meng Gao, Weili Wang, Wei Li, Sijin Liu, Zhifang Chai, Ruibin Li

**Affiliations:** 1 State Key Laboratory of Radiation Medicine and Protection, School for Radiological and Interdisciplinary Sciences (RAD-X), Collaborative Innovation Center of Radiological Medicine of Jiangsu Higher Education Institutions, Soochow University, Suzhou, 215123 Jiangsu China; 2 State Key Laboratory of Environmental Chemistry and Ecotoxicology, Research Center for Eco-Environmental Sciences, Chinese Academy of Sciences, 18 Shuangqing Road, Beijing, 100085 China

**Keywords:** Cell death, Inflammation, Nanotoxicology

## Abstract

Sustainable developments of nanotechnology necessitate the exploration of structure-activity relationships (SARs) at nano-bio interfaces. While ferroptosis may contribute in the developments of some severe diseases (e.g., Parkinson’s disease, stroke and tumors), the cellular pathways and nano-SARs are rarely explored in diseases elicited by nano-sized ferroptosis inducers. Here we find that WS_2_ and MoS_2_ nanosheets induce an iron-dependent cell death, ferroptosis in epithelial (BEAS-2B) and macrophage (THP-1) cells, evidenced by the suppression of glutathione peroxidase 4 (GPX4), oxygen radical generation and lipid peroxidation. Notably, nano-SAR analysis of 20 transition metal dichalcogenides (TMDs) disclosures the decisive role of surface vacancy in ferroptosis. We therefore develop methanol and sulfide passivation as safe design approaches for TMD nanosheets. These findings are validated in animal lungs by oropharyngeal aspiration of TMD nanosheets. Overall, our study highlights the key cellular events as well as nano-SARs in TMD-induced ferroptosis, which may facilitate the safe design of nanoproducts.

## Abstract (web summary)

It is unclear whether 2D metal dichalcogenides (TMD) alone can cause ferroptotic cell death. Here, the authors show TMD nanosheets induced ferroptosis in mammalian cell lines and in a mouse model after aspiration of TMD materials into lungs, causing ferroptotic cell death.

## Introduction

As physicochemical properties of engineered nanomaterials (ENMs) were found to significantly impact their toxicities, structure-activity relationships (SARs) of ENMs have been highly underlined in nanobiology research^1^. Some progresses on nano-SARs have been made in ENM-induced apoptosis^2^, carcinogenesis^3^, autophagic^4^, and inflammatory effects^5^. For instance, diameter and rigidity of carbon nanotubes are critical characteristics in mesothelial injury and carcinogenesis^3^; carbon radicals of graphene oxides are able to induce lipid peroxidation and cell deaths^6^. Recently, two-dimensional (2D) transition metal dichalcogenides (TMDs) as an atomic-layer semiconductor were found to display unique surface activities^7^ and arouse great enthusiasm for their biological effects. The surface area, active edge sites, and aggregation states were found to impact the hazard effects of TMDs in cells and animals^8–11^. For instance, Wang et al. discovered that aggregated MoS_2_ induced proinflammatory cytokine release in cells and acute lung inflammation in mice, whereas dispersed MoS_2_ had little effect^9^.

Beside of the above hazard effects, ferroptosis is a newly recognized iron-dependent cell death mechanism, characterized by generation of lipid peroxidation^12^. This unique cell death mechanism closely relates to some serious diseases such as Parkinson’s disease^12,13^, stroke^14,15^ and tumors^16^. Currently, silica nanoparticles are the only non-iron materials that are able to induce ferroptosis in starved M21 cancer cells and cancer-bearing mice^17^. However, the nano-SARs involved in ferroptosis are unexplored, and other nano inducers are rarely reported.

Herein, WS_2_ and MoS_2_ nanosheets are found to induce ferroptosis in epithelial (BEAS-2B) and macrophage (THP-1) cells, which are the first port of entry for exposed fine particulates in vivo. To explore the nano-SARs involved in ferroptosis, five 2D TMDs are prepared by exfoliating nanosheets from bulk materials of WS_2_, MoS_2_, WSe_2_ and MoSe_2_ and BN. The toxicity pathways of TMDs are deciphered by examining the molecular initiating events (MIEs) at nano-bio interfaces. We are therefore able to identify the key physicochemical properties that are responsible for ferroptotic cell deaths. The identified nano-SARs are utilized for biocompatible functionalization of WS_2_ and MoS_2_ nanosheets. Considering the potential inhalation exposure risks of nanoproducts such as lubricants, semiconductors, and surface coatings, the in vitro results are further validated in animal lungs by oropharyngeal aspiration of TMD materials.

## Results

### Assessments of cytotoxicity by 2D TMDs

**Fig. 1:**
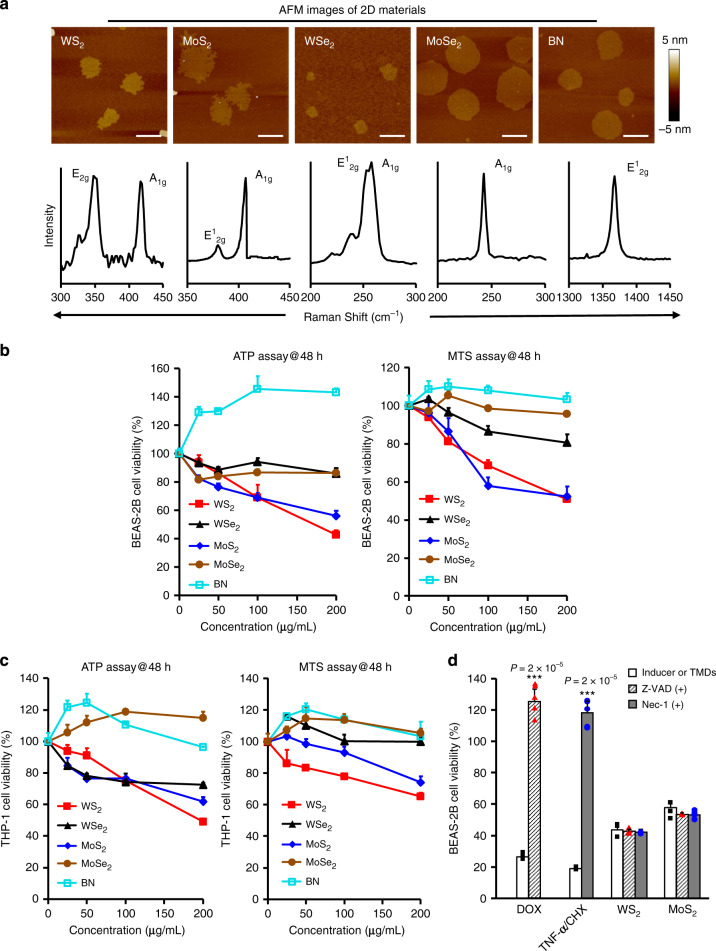
Characterization of 2D TMDs and their cytotoxicity assessments. **a** Representative AFM images and Raman spectra of 2D TMDs. A drop of 2D TMDs suspension (50 μg/mL) was placed on a mica plate, and then dried in vacuum drying oven for AFM observation (scale bar, 100 nm). 2D TMDs stock solutions (5 mg/mL) were freeze-dried to form powder for Raman spectra detection. Shown are the representative images from three independent experiments. Cell viability test in **b** BEAS-2B and **c** THP-1 cells. BEAS-2B and THP-1 cells were exposed to 2D TMDs at 0–200 μg/mL for 48 h. The cell viability was examined by detecting luminescence and absorbance of substrates in ATP and MTS assays (*n* = 3 biologically independent cell samples). Data are presented as mean values ± SD. **d** Effects of apoptosis and necrosis inhibitors on MoS_2_ and WS_2_ induced cytotoxicity. BEAS-2B cells pretreated with 50 μM Nec-1, 20 μM Z-VAD-FMK for 1 h were exposed to 2 μg/mL DOX, formula of 100 ng/mL TNF-α and 1 μg/mL CHX, 200 μg/mL WS_2_ and MoS_2_ for 48 h to determine cell viability by ATP assay (*n* = 3 biologically independent cell samples). Data are presented as mean values ± SD. ***p < 0.001 compared to inducer or TMD treatments by two-tailored Student *t*-test.

To prepare 2D materials, bulk WS_2_, MoS_2_, WSe_2_, MoSe_2_, and BN materials were ground and dispersed in aqueous Pluronic F68 solutions for sufficient ultrasonication to exfoliate nanosheets. The suspensions were ultra-centrifuged to collect the supernatants containing monolayer or few-layer nanosheets for sufficient dialysis. The purified 2D materials were characterized by atomic force microscope (AFM), Raman spectrometer, Zeta potential and hydrodynamic analyzer for structure interpretations. AFM images showed that all five 2D materials had irregular planner structures with diameters and heights at 16–264 nm and 0.3–1.8 nm, respectively (Fig. 1a, Supplementary Fig. 1A). The 2D planner structures were further evidenced by the Raman characteristic peaks, e.g., E_2g_ or A_1g_ peaks of WS_2_ (~347, 416 cm^−1^)^18^, E^1^_2g_ or A_1g_ peaks of MoS_2_ (~380, 407 cm^−1^)^19^ and WSe_2_ (~252, 257 cm^−1^)^20^, A_1g_ peak of MoSe_2_ (~242 cm^−1^)^21^ and E^1^_2g_ peak of BN (~1367 cm^−1^)^22^. The surface charges and hydrodynamic sizes were assessed in water and cell culture media (Supplementary Table 1). All five materials showed negative surface charges with zeta potentials in the range of −5.8 to −25.9 mV in water, and they had similar hydrodynamic sizes in DI water (90–128 nm), RPMI 1640 (96–134 nm) and bronchial epithelial growth medium (BEGM, 92–135 nm). As shown in Supplementary Fig. 1B, except for WSe_2_, other four materials exhibited good suspension stability in RPMI 1640 for a period of 48 h incubation.

Then BEAS-2B and THP-1 cells were exposed to the 2D materials for assessments of their bio-effects. First, we assessed the impacts of 2D materials on cell proliferation by measuring the endogenous ATP levels. As shown in Fig. 1b, c and Supplementary Fig. 2, ATP assay suggested that WS_2_ and MoS_2_ induced dose- and time-dependent cell death in BEAS-2B and THP-1 cells, whereas BN, MoSe_2_, and WSe_2_ had little effect on cell viability. After 48 h exposure at the highest dose of 200 μg/mL, WS_2_ and MoS_2_ induced 57.3 and 43.8% cell deaths, respectively. This cytotoxic effect could be further validated by examining the activities of dehydrogenases in cellular metabolic process via MTS assay. Then we attempted to differentiate the cell death types by dynamically visualizing cell morphology changes under a time-lapse microscope. Doxorubicin (DOX) was used as apoptosis inducer^23^ and a formula of TNF-α and cycloheximide (CHX) was used as necrosis inducer^24^. The cell growth movie showed that WS_2_ treated cells preferred to recruit around particle agglomerates, fuse together and lyse (Movie S1), which is similar to the death stages in necrosis (Movie S2) but different to apoptosis (Movie S3). However, necrostatin-1 (Nec-1) as a conventional inhibitor of necrosis was failed to ameliorate MoS_2_/WS_2_ induced cytotoxicity. Apoptosis inhibitor (Z-VAD-FMK) neither had effects (Fig. 1d). We therefore speculated that a different cytotoxicity mechanism may be involved in MoS_2_ and WS_2_ induced cell deaths.

### Endocytosis was required in TMD-induced cell deaths

**Fig. 2:**
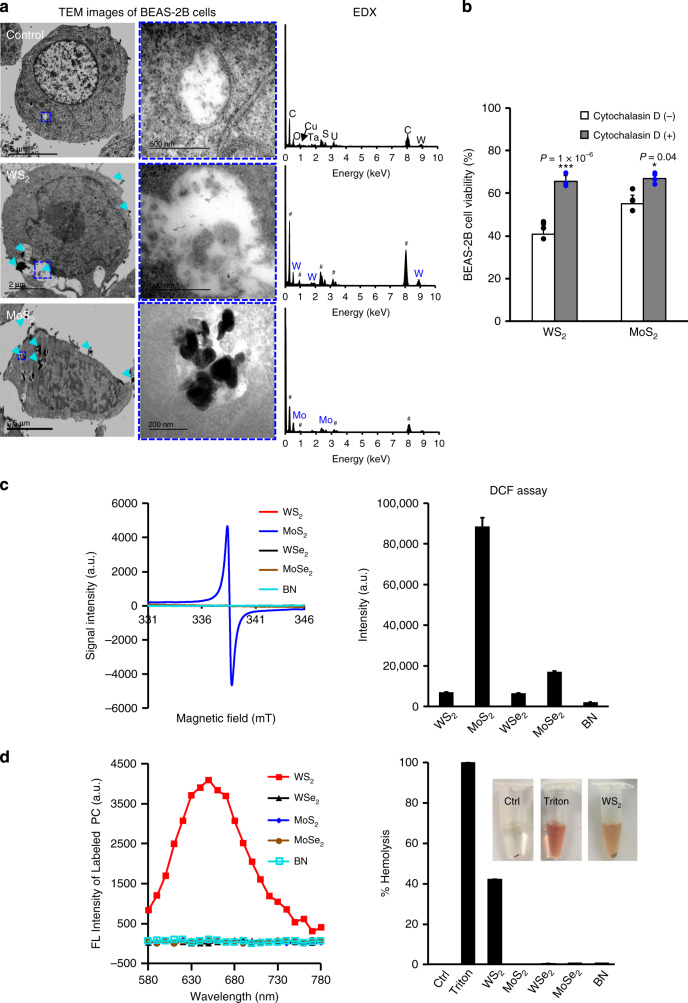
Exploration of key TMD properties responsible for cytotoxicity. **a** Representative TEM images of TMD distribution in cells. BEAS-2B cells treated by 100 μg/mL WS_2_ and MoS_2_ for 24 h were collected to fix, stain and prepare slides for TEM observation. Shown are the representative images from three independent cell samples. The cyan arrows represent the TMDs. Symbol (^#^) represents background elements. **b** Impacts of endocytosis inhibitor on cytotoxicity of WS_2_ and MoS_2_. BEAS-2B cells pretreated with 5 μg/mL CD for 2 h were exposed to 200 μg/mL WS_2_ and MoS_2_ for cell viability test after 48 h incubation (*n* = 3 biologically independent cell samples). Data are presented as mean values ± SD. *p < 0.05, ***p < 0.001 compared to TMD treatments by two-tailored Student *t*-test. **c** Assessment of surface radicals on TMD surfaces (*n* = 3 independent samples). All 2D materials were subjected to EPR measurement at a *g* value of 2.003133 (left). The oxidation potentials were assessed by detection of the fluorescence of H_2_DCF after 2 h incubation with 250 μg/mL of TMDs (right). Data are presented as mean values ± SD. **d** Interactions between TMDs and lipid layers (*n* = 3 independent experiments). The fluorescence of Nile red labeled lipids were detected in supernatants resulting from DSPC liposomes (1 mg/mL) reacted with 200 μg/mL TMDs for 4 h (left). Hemolysis assay was performed by incubation of fresh mouse blood cells with 100 μg/mL 2D TMDs in PBS to detect the absorbance of released hemoglobin in supernatants (right). Data are presented as mean values ± SD.

To decipher the detailed mechanism, we first examined whether the metal ions released from MoS_2_ and WS_2_ were responsible for their cytotoxicity. Viability test of BEAS-2B cells exposed to the supernatants and pellets of TMD suspensions showed that particle dissolution had little effects to cell proliferation (Supplementary Fig. 3). We then visualized the cellular distribution of 2D TMD nanosheets by transmission electron microscope (TEM). As shown in Fig. 2a, suspected TMD agglomerates with high electron densities could be visualized in the vesicular structures as well as extracellular regions. Energy Dispersive X-ray (EDX) analysis confirmed the presence of W and Mo elements in these agglomerates. The cellular distribution was further confirmed by confocal microscopy imaging of fluorescein isothiocyanate (FITC)-labeled WS_2_ and MoS_2_ nanosheets in BEAS-2B cells. As shown in Supplementary Fig. 4, the labeled nanosheets had a colocalization with lysosomes lighted by a lysotracker. Next, we questioned whether the cytotoxicity of WS_2_ and MoS_2_ resulted from cellular internalized or extracellular nanosheets. Cytochalasin D, an inhibitor of cell actin monomer polymerization, was used for the blockage of nanoparticle endocytosis. As shown in Fig. 2b, cytochalasin D pretreatment significantly reduced the toxic effects of WS_2_ and MoS_2_ in BEAS-2B cells. These data suggested that endocytosis was required in cell deaths induced by WS_2_ and MoS_2._

Based on these observations, we speculated that lysosomal compartment is likely to be the primary scene of initial toxicity events. To decipher the MIEs in lysosomes, we comprehensively examined the physicochemical property changes (surface area, oxidation potential, surface charge and dissolution) of TMD nanosheets in phagolysosomal simulated fluids (PSF) as well as their interactions with lysosome compartments. We identified strong oxidation capability and phospholipid-binding affinity on MoS_2_ and WS_2_ surfaces, respectively. As shown in Fig. 2c, unpaired electrons of Mo atoms could be detected by the peaks at 338 mT on the spectra of electron paramagnetic resonance (EPR). MoS_2_ therefore exhibited strong oxidation potentials, evidenced by the increased fluorescence of oxidized dihydrodichlorofluorescein (H_2_DCF) by MoS_2_ nanosheets in PSF. In addition, strong adsorption of phospholipids was detected on WS_2_ surfaces using Nile Red labeled phosphatidylcholine (PC) liposomes, which may be attributed to the high binding affinity of unsaturated W elements with phosphine to form W–O–P bonds^25^. This interaction was further confirmed by a hemolysis analysis where WS_2_ could induce 42% hemoglobin release from damaged red blood cells (Fig. 2d).

### Deciphering ferroptosis pathways of MoS_2_ and WS_2_ nanosheets

**Fig. 3:**
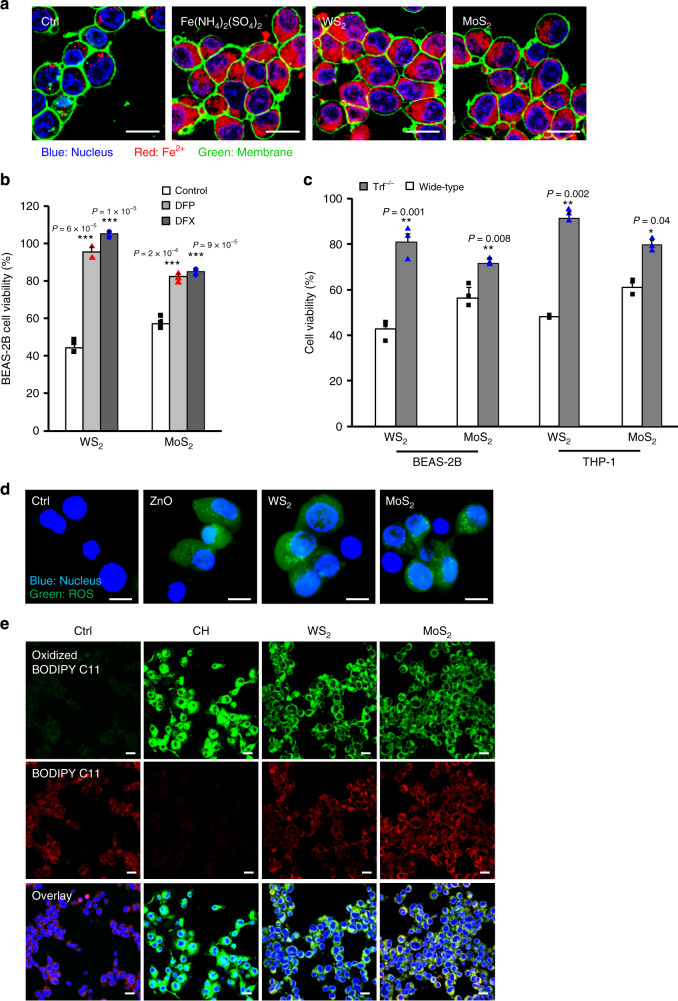
Biomarkers of ferroptosis in TMD-exposed cells. **a** Representative images of Fe^2+^ in cells by confocal microscope. BEAS-2B cells treated by 100 μM Fe(NH_4_)_2_(SO_4_)_2_, 200 μg/mL WS_2_ and MoS_2_ were stained by FeRhoNox (red) to visualize the cellular distribution of Fe^2+^. Hoechst 33342 and WGA were used to stain nuclei (blue) and cell membrane (green), respectively (scale bar: 10 μm). Shown are the representative images from three independent cell samples. **b** Effects of Fe^2+^ chelators on WS_2_ and MoS_2_ induced cytotoxicity (*n* = 3 biologically independent cell samples). BEAS-2B cells pretreated by 2 mM DFP or 0.4 mM DFX were exposed to 200 μg/mL WS_2_ and MoS_2_ and examine cell viability after 48 h incubation. Data are presented as mean values ± SD. ***p < 0.001 compared to untreated cells by two-tailored Student *t*-test. **c** Comparison of cell viabilities in wide-type and TfR-KD cells, cell viabilities in wide-type and TfR-KD cells exposed to 200 μg/mL WS_2_/MoS_2_ for 48 h were assessed by ATP assay (*n* = 3 biologically independent cell samples). Data are presented as mean values ± SD. **p < 0.01, ***p < 0.001 compared to wide-type cells by two-tailored Student *t*-test. **d** Representative images of ROS and **e** lipid peroxidation in BEAS-2B cells. After 12 incubation with 200 μg/mL WS_2_ and MoS_2_ for 12 h, BEAS-2B cells were stained with H_2_DCF-DA (scale bar: 10 μm) and Image-iT lipid peroxidation staining kit (scale bar: 10 μm) for confocal observation of nuclei (blue), reduced substrate (red), and oxidized substrate (green). 10 μM CH were used as positive controls. Shown are the representative images from three independent cell samples.

Considering lysosome is an important depository for large amounts of redox-active iron, ferrous ion release was examined in TMD-treated cells. Non-fluorescent FeRhoNox-1 was used to detect labile Fe^2+^ in cells as this substrate can specifically react with Fe^2+^ to emit intense fluorescence at 575 nm^26^. As shown in Fig. 3a, in contrast to the punctate distribution of red dots in control cells, increased Fe^2+^ leakage was confirmed in WS_2_ and MoS_2_ treated cells, displaying diffused distribution pattern of FeRhoNox-1 similar to Fe(NH_4_)_2_(SO_4_)_2_ treatment (positive control). Since lysosomal iron release have been reported as the key upstream signals of ferroptosis, we hypothesized that WS_2_ and MoS_2_ nanosheets were able to induce ferroptotic cell death. To demonstrate this, we first examined whether the cytotoxicity of WS_2_ and MoS_2_ nanosheets was dependent on lysosomal release of Fe^2+^ by comparing cell viabilities in normal and iron-chelator treated cells. Two clinically used chelators including deferiprone (DFP) and deferasirox (DFX) were exploited to chelate cytosolic iron. As shown in Fig. 3b, DFP and DFX pretreatments significantly ameliorated the toxic effects of MoS_2_ and WS_2_, and rescued more than 27.9% cells, suggesting an iron-dependent cell death pathway. Since transferrin receptors (TfR) serving as the dominant transporter of iron in cell metabolism is very relevant to ferroptosis^27^, TfR knockdown (TfR-KD) is a reliable method to examine ferroptotic cell death^28,29^. The iron-dependent cytotoxic effect of MoS_2_ and WS_2_ was also validated in TfR-KD cells. As shown in Fig. 3c, Both MoS_2_ and WS_2_ nanosheets induced less cell deaths in TfR-KD cells than those in wide-type (WT) cells. These results well supported our claim on the ferroptosis effect of MoS_2_ and WS_2_.

Since redox-active ferrous ions could catalyze the generation of reactive oxygen species (ROS) via Fenton reaction, we reasoned that TMD-induced lysosomal release of Fe^2+^ might subsequently result in ROS generation. To demonstrate this, nanoparticle treated BEAS-2B cells were stained by nonfluorescent 2′,7′-Dichlorofluorescin diacetate (H_2_DCF-DA), which could be oxidized into dichlorofluorescein (DCF) by ROS and emit strong green fluorescence at 529 nm. Confocal microscopy images showed that intensive DCF fluorescence could be visualized in WS_2_ and MoS_2_ treated cells, indicating massive ROS generation, whereas DFP/DFX pretreatments could dramatically reduce this effect (Fig. 3d). N-acetyl-cysteine (NAC) and 6-hydroxy-2,5,7,8-tetramethylchroman-2-carboxylic acid (Trolox) as scavengers of ROS could completely eliminate the hazard effects of MoS_2_/WS_2_ in BEAS-2B cells (Supplementary Fig. 5). Consequently, we examined a ROS-induced lethal signal of ferroptosis, lipid peroxidation in BEAS-2B cells. Assessments of lipid peroxidation in live cells could be achieved by detecting the oxidation of BODIPY 581/591 C11 reagent, which displays a shift of fluorescence emission peaks from ~590 nm (red) to ~510 nm (green) upon oxidation by lipid hydroperoxides. Cumene hydroperoxide (CH) was included as a positive control. As shown in Fig. 3e, MoS_2_ and WS_2_ treatment enabled remarkable lipid peroxidation in BEAS-2B cells signified by a ratiometric increment of green vs. red fluorescence, whereas a majority of cells emit uniform red fluorescence of BODIPY 581/591 C11 in untreated cells. As a result, substantial dead cells could be visualized by LIVE/DEAD staining in MoS_2_ and WS_2_ treatments, displaying robust red fluorescence of Ethidium homodimer-1 (EthD-1) that is capable of passing through damaged cell membranes and binding with DNA molecules (Supplementary Fig. 6). The green fluorescence of calcein-AM indicated viable cells with intact cell membranes.

**Fig. 4:**
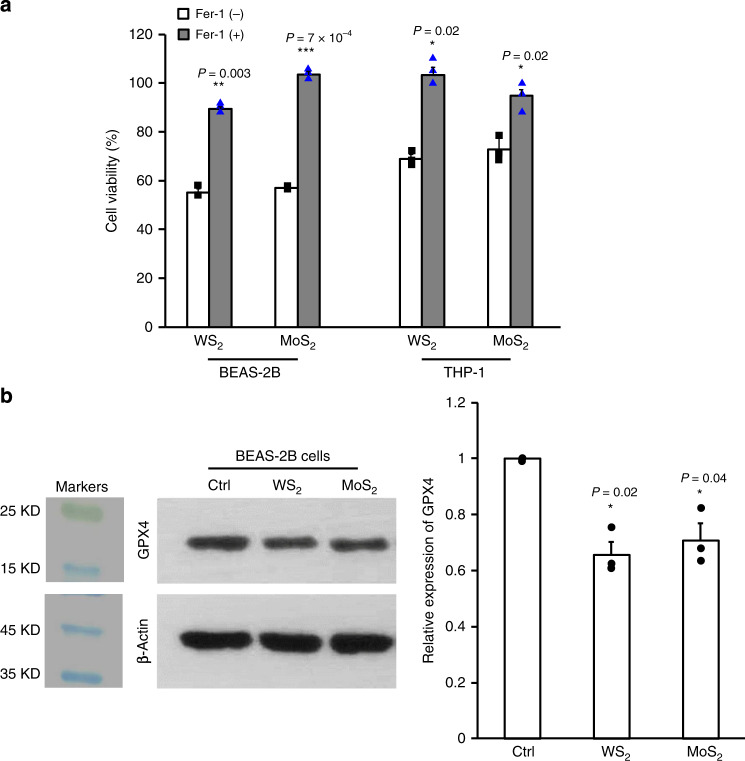
Impacts of Fer-1 and GPX4 expression in TMD-treated cells. **a** Effects of Fer-1 on MoS_2_ and WS_2_ induced cytotoxicity (*n* = 3 biologically independent cell samples). BEAS-2B and THP-1 cells were pretreated by 10 μM Fer-1 for 1 h, followed by 48 h incubation with 200 μg/mL WS_2_ and MoS_2_ (*n* = 3). MTS assay was used to assess the impacts of Fer-1 on WS_2_/MoS_2_-induced cell deaths. Data are presented as mean values ± SD. **p < 0.01 and ***p < 0.001 compared to cells without Fer-1 pretreatment by two-tailored Student *t*-test. **b** Assessment of GPX4 expression in BEAS-2B cells. After exposure to 200 μg/mL WS_2_ and MoS_2_ for 48 h, BEAS-2B cells were collected, washed and lyzed for Western blotting assay (left panel). The relative expression of GPX4 was assessed by the optical density of each band on representative Western blotting images (right panel, *n* = 3 independent experiments). Data are presented as mean values ± SD. *p < 0.05, compared to Ctrl cells by two tailored Student *t*-test.

To further confirm the ferroptosis mechanism, we examined the impacts of a ferroptosis inhibitor, ferrostatin-1 (Fer-1) on WS_2_ and MoS_2_ induced cell deaths. As shown in Fig. 4a, Fer-1 pretreatment could dramatically improve cell viabilities (24-47% increments) in BEAS-2B and THP-1 cells exposed to WS_2_ and MoS_2_. A reduction of glutathione peroxidase 4 (GPX4) in response to WS_2_ and MoS_2_ nanosheets was confirmed by Western blotting (Fig. 4b). Immunoblotting to examine the abundance of GPX4 in BEAS-2B extracts demonstrated that WS_2_ and MoS_2_ treatments suppressed >30% expression of GPX4 in cells. These results allowed us to decipher the cascaded signaling pathways of TMD-induced ferroptosis. In brief, TMD nanosheets could be endocytosed into lysosomes for digestion. Lysosomal processing of the unsaturated metal atoms on WS_2_ and MoS_2_ surfaces may result in lysosomal membrane changes via phospholipid extraction and oxidation damages, respectively. The damaged lysosomal membranes allowed Fe^2+^ leakage into cytoplasm to serve as catalysts in Fenton reactions and trigger substantial ROS generation, causing lipid peroxidation, membrane deconstruction and cell death.

### Exploration of nano-SARs in ferroptosis

Since the active Mo or W atoms resulting from surface vacancies were found to take part in the MIEs of ferroptosis, we hypothesized that the surface vacancy may be the key physicochemical property responsible for the ferroptosis effect of MoS_2_ and WS_2_ nanosheets. To demonstrate this hypothesis, we prepared 20 derivatives of TMD nanoparticles to examine the impacts of vacancy on cell viability as nano-SARs are often determined by in silico analysis of quantitative descriptors of intrinsic properties and biological receptors acquired from sufficiently large databases of nanomaterials^30,31^. The vacancy densities of TMDs were assessed by surface atom ratios of S/Se vs. W/Mo^32,33^. As shown in Table 1, these nanoparticles had different compositions, sizes, surface charges, and surface atom ratios. We examined the correlations between cell viabilities and physicochemical properties of TMDs. Pearson correlation analysis showed that surface atom ratio (vacancy density) had strong correlation with cell viability, displaying a correlation factor of |r| at 0.89. This result indicated that surface vacancy may be the critical property dictating the ferroptosis effect induced by WS_2_ and MoS_2_ nanosheets.

**Table 1:** Correlation analysis between cell viability and physicochemical property.

TMD		Physicochemical properties					Cell viability (%)
		Thickness (nm)	Diameter (nm)	Hydrodynamic size (nm)	Zeta potential (nm)	Surface atom ratio^a^	
WS_2_	1	211 ± 8	384 ± 9	189.7 ± 0.4	−20.7 ± 0.4	1.77	108.4
	2	90 ± 3	178 ± 5	139.5 ± 3.2	−24.5 ± 1.5	1.82	117.4
	3	40 ± 2	108 ± 3	120.2 ± 0.4	−24.7 ± 1.3	1.75	108.7
	4	2 ± 1	10 ± 2	94 ± 2	−6 ± 0.3	1.21	54.6
	5	2 ± 1	12 ± 1	96 ± 1	−7.2 ± 2	1.77	89.2
MoS_2_	1	153 ± 6	407 ± 2	308.7 ± 1.8	−25.4 ± 1.2	1.71	113.9
	2	79 ± 3	199 ± 5	143.9 ± 0.2	−24.8 ± 0.8	1.70	114.2
	3	49 ± 3	175 ± 1	115.3 ± 0.9	−27.5 ± 0.4	1.53	91.5
	4	3 ± 1	9 ± 3	91 ± 1	−18.6 ± 0.3	1.31	57.3
	5	3 ± 1	10 ± 2	93 ± 2	−17.5 ± 1.0	1.65	101.2
WSe_2_	1	202 ± 8	304 ± 6	204 ± 4	−20.7 ± 0.4	1.65	105.3
	2	140 ± 4	92 ± 2	146 ± 4	−27.8 ± 0.7	1.71	116.9
	3	42 ± 8	78 ± 4	150 ± 5	−20.3 ± 0.2	1.60	99.9
	4	16 ± 4	62 ± 6	106 ± 0.5	−23.5 ± 0.1	1.61	96.2
	5	2 ± 1	14 ± 3	94 ± 1	−24.2 ± 0.3	1.64	99.4
MoSe_2_	1	160 ± 2	232 ± 2	349 ± 9	−21.5 ± 0.3	1.73	100.5
	2	91 ± 7	113 ± 9	152 ± 1	−20.9 ± 0.1	1.80	112.9
	3	39 ± 7	73 ± 9	145 ± 3	−20.4 ± 0.2	1.81	115.3
	4	21 ± 4	50 ± 1	120 ± 5	−20.2 ± 0.2	1.71	98.4
	5	2 ± 1	16 ± 4	123 ± 2	−18.7 ± 0.4	1.68	92.8
Correlation factor		0.51	0.48	0.38	−0.64	0.89	NA

^a^Represents the ratio of S/Se vs. W/Mo determined by X-ray photoelectron spectroscopy (XPS). Cell viability data were acquired in BEAS-2B cells exposed to 200 μg/mL for 48 h by MTS assay. Data are presented as mean values ± SD. Replicate numbers: thickness (*n* = 50), diameter (*n* = 50), hydrodynamic size (*n* = 10), Zeta potential (*n* = 10), surface atomic ratio (*n* = 3), and cell viability (*n* = 3).

**Fig. 5:**
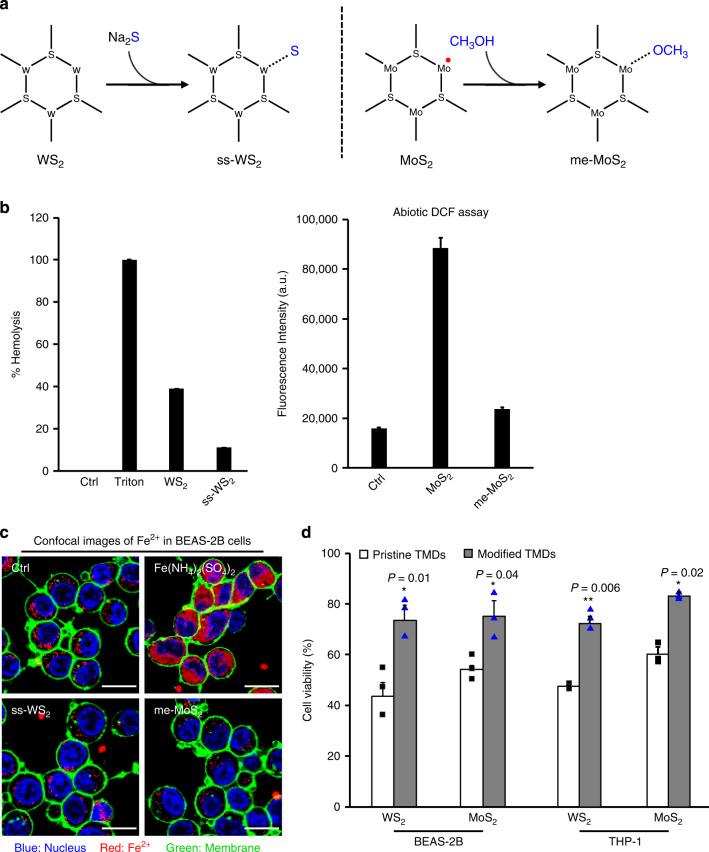
Safe design of WS_2_ and MoS_2_ by surface passivation. **a** Schematic images to passivate nanosheet surfaces. **b** Assessment of surface passivation by hemolysis or abiotic DCF assay (*n* = 3 independent experiments). Data are presented as mean values ± SD. **c** Representative images of lysosomal release of Fe^2+^ and **d** cell viability test in cells exposed to modified TMDs. To assess the impacts of surface passivation, BEAS-2B and THP-1 cells were exposed to 200 μg/mL nanosheets for 48 h. The treated cells were subject to MTS assay or FeRhoNox staining for confocal imaging (*n* = 3 biologically independent cell samples). Scale bar represents 10 μm. Cell viability data are presented as mean values ± SD. *p < 0.05, **p < 0.01 compared to the pristine TMD-treated cells by two-tailored Student *t*-test.

This nano-SAR information was further validated by engineering design of WS_2_/MoS_2_ for vacancy healing (Fig. 5a). Specifically, we found Na_2_S treatment is able to reduce phospholipid-binding activity of WS_2_ while methanol is capable of quenching surface radicals on MoS_2_. These functionalized nanosheets were denoted as ss-WS_2_ and me-MoS_2_. We exploited high resolution (HR) TEM to visualize the vacancies on pristine and functionalized MoS_2_ and WS_2_ nanosheets. As shown in Supplementary Fig. 7, while substantial single vacancies and linear surface vacancies could be identified on the surfaces of WS_2_ and MoS_2_ nanosheets (yellow circles), surface passivation is an effective strategy for vacancy healing. Before biocompatibility tests, we first examined whether the surface modifications may affect the lubricating properties of TMDs as they are often used as lubricant additives. As shown in Supplementary Fig. 8, Na_2_S and methanol treatments had negligible effects on the friction coefficients of WS_2_ and MoS_2_, respectively. We therefore thought the surface passivation did not affect the utility of TMDs as lubricants. In addition, surface passivation could significantly reduce the interactions between WS_2_ and membrane lipids as well as the radicals on MoS_2_ surfaces (Fig. 5b and Supplementary Fig. 9). We used isolated lysosomes from BEAS-2B cells to examine the oxidation capability of MoS_2_ and me-MoS_2_ by the lipid peroxidation staining kit. As shown in Supplementary Fig. 10, MoS_2_ induced significant oxidation of isolated lysosomes emitting strong fluorescence of oxidized BODIPY 581/591 C11, whereas surface passivation could fully ameliorate this effect. Biocompatibility test in cells showed that the modified nanosheets had similar cellular uptake to pristine TMDs (Supplementary Fig. 11), but less effects on lysosomal damage evidenced by diminishing Fe^2+^ release in cytoplasm (Fig. 5c) as well as ferroptotic cell deaths (Fig. 5d). All these results well demonstrate our hypothesis on the role of surface vacancies in TMD-induced ferroptosis. The Na_2_S and methanol treatments could be considered as safe design approaches for WS_2_ and MoS_2_ nanosheets because these passivation methods could significantly reduce the cytotoxic effects of two nanosheets without affecting their utilities as lubricants.

### Validation of TMD-induced ferroptosis in vivo

**Fig. 6:**
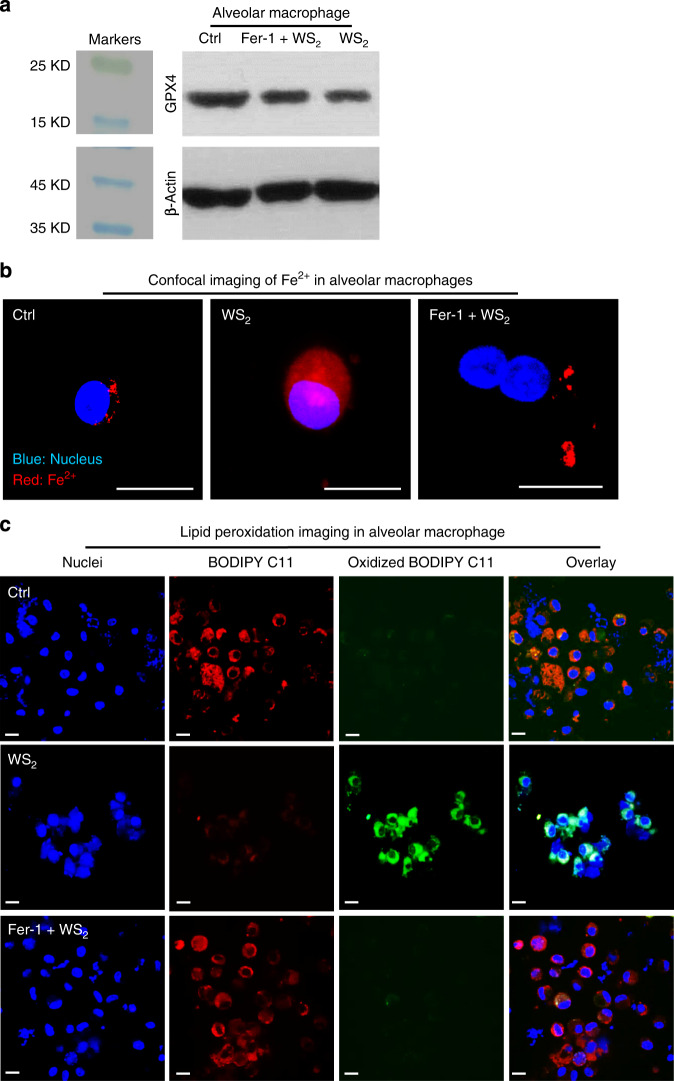
Validation of ferroptosis in alveolar macrophages from lung tissue. **a** Representative Western blotting images of GPX4 from three independent experiments, **b** representative images of lysosomal release of Fe^2+^, and **c** lipid peroxidation in alveolar macrophages from four independent views. Animals pretreated with or without 50 μg/Kg Fer-1 for 2 h were exposed to 1 mg/Kg WS_2_ for 40 h (*n* = 6 mice). Then the BALF of animals were collected to extract alveolar macrophages. The cells were divided into three portions. One was lysed for Western blotting analysis, and the other two were stained by Image-iT lipid peroxidation staining kit (scale bar: 10 μm) to visualize nuclei (blue), reduced substrate (red) and oxidized substrate (green), or FeRhoNox-1 (scale bar: 10 μm) to observe lysosomal release of Fe^2+^.

The ferroptosis effects as well as the identified nano-SAR were finally validated in animals. From the perspective of inhalation exposure risks of 2D TMDs in real scenarios, we examined the pulmonary toxicity of WS_2_, MoS_2_, MoSe_2_, ss-WS_2_, and me-MoS_2_ by oropharyngeal aspiration. Quartz was included as a positive control. Following 40 h exposure, mice were sacrificed to collect bronchoalveolar lavage fluid (BALF) to examine the effects of TMDs on cells and cytokines. First of all, we examined GPX4 expression, lysosomal release of Fe^2+^ and lipid peroxidation to validate the ferroptosis mechanism. As shown in Fig. 6a, reduction of GPX4 was identified in alveolar macrophages extracted from animal lungs exposed to WS_2_ nanosheets. Meanwhile, WS_2_ treatments led to significant Fe^2+^ leakage in alveolar macrophages by FeRhoNox-1 staining, displaying diffused red fluorescence in whole cytoplasm (Fig. 6b). Subsequently, the released Fe^2+^ could elicit lipid peroxidation, evidenced by a majority of alveolar macrophages displaying strong green fluorescence of oxidized BODIPY C11 (Fig. 6c). Notably, Fer-1 treatment could ameliorate the impacts of WS_2_ on GPX4 expression, and suppressed lysosomal release of Fe^2+^ and lipid peroxidation in alveolar macrophages. Surface passivation could also attenuate these key signals of ferroptosis in alveolar macrophages (Supplementary Fig. 12). These data showed that the impact of the TMDs on pulmonary alveolar macrophages duplicated the results seen in tissue culture cells.

**Fig. 7:**
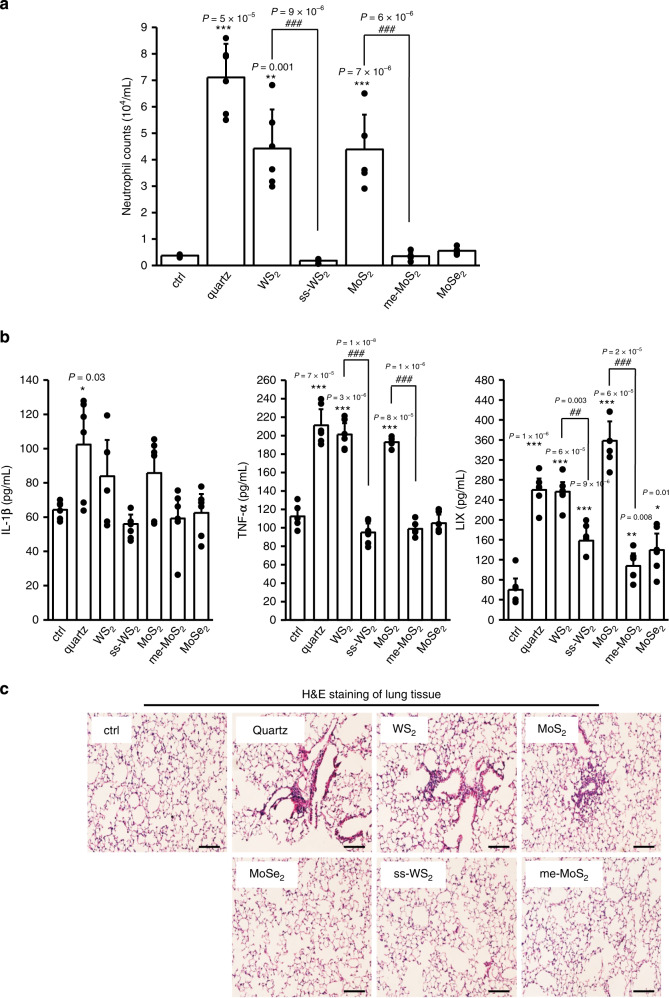
Assessment of the effects of surface passivation in mouse lungs. **a** Neutrophil counts in BALF. Animals were exposed to 1 mg/Kg pristine or surface modified TMDs by oropharyngeal aspiration (*n* = 6 mice). After 40 h, the animals were sacrificed and alveolar macrophages in BALF of mouse lungs were collected for overnight incubation. Immune cells in BALF were concentrated on glass slides by cytospin, fixed and stained by Quick-Diff for cell counting. Neutrophil count data are presented as mean values ± SD. **b** Pro-inflammatory cytokine production in BALF and **c** representative H&E staining images of lung tissues from six biologically independent samples (scale bar: 100 μm). Pro-inflammatory cytokines were determined by ELISA. Cytokine data are presented as mean values ± SD. *p < 0.05, **p < 0.01, ***p < 0.001 compared to vehicle controls, ^#^*p* < 0.05, ^##^*p* < 0.01, ^###^*p* < 0.001 compared to WS_2_ and MoS_2_ by two-tailored Student *t*-test.

We also questioned whether the ferroptosis effect of TMDs would result in pro-inflammatory effects in mouse lungs. As shown in Fig. 7a, quartz and pristine TMDs induced significantly higher levels of neutrophil recruitment in BALF, compared to the neutrophil counts in ss-WS_2_ and me-MoS_2_ treatments. The pro-inflammatory response of pristine TMDs in the BALF was reflected in the increases of cytokines including LIX, TNF-α, and IL-1β. By contrast, surface passivation could significantly reduce the inflammatory cytokine production (Fig. 7b). Consistently, while me-MoS_2_ and ss-WS_2_ exhibited negligible inflammatory effects in focal pulmonary infiltrates by hematoxylin and eosin (H&E) staining, pristine MoS_2_ and WS_2_ elicited significant lung inflammation, displaying increased nucleus densities due to immune cell recruitments (Fig. 7c). All these animal results well confirmed our in vitro findings.

## Discussion

2D TMDs consist of a monolayer or few-layer covalently bonded chalcogen and metal atoms. Unlike 2D graphene materials, the transition metal and dichalcogenide atoms of TMDs possess abundant electrons in *d* or *f* orbitals, which may confer intriguing surface properties, such as high photoluminescence quantum yield^34,35^, sizeable bandgap^36,37^, valley-selective circular dichroism^38,39^ and strong photocurrent responses^40,41^. The industrial uses of 2D TMDs have led to increasing exposure risks to humans as well as substantial concerns on their biosafety. Since 2D TMD materials have exhibited many intriguing surface chemistries and justified their potential applications in many fields, their interactions with biological systems have been underlined^42^. We therefore proposed this study to investigate the hazard effects and nano-SARs of TMDs in mammalian cells. Compared to other nano-bio studies on 2D TMDs, our study made two findings: (i) MoS_2_ and WS_2_ were able to induce ferroptosis in cells and animal lungs; (ii) the vacancy on nanosheet surfaces was responsible for the ferroptosis cell deaths. Beside of the direct impacts of surface vacancy on cell viability, inhaled TMD nanosheets may escape the clearance by mucociliary escalator, deposit in pulmonary alveoli and interact with lung surfactants and proteins to form bio-corona structures^43^. The adsorption of immunoglobulins, complement factors, lipids and coagulation proteins on TMD surfaces may lead to the recognition and capture by immune cells in vivo, eliciting rapid clearance, and significant immunotoxicity^44,45^. In contrast, formation of protein corona in vitro may reduce the cellular internalization of nanoparticles and ameliorate cytotoxicity due to improved biocompatibility^43,46^.

Recently, a few routine hazard signals widely reported in a majority of engineered nanomaterials have been identified in TMD-treated cells or animals. For instance, MoS_2_ nanosheets were found to induce reactive oxidative species and cell deaths in A549 cells^8^. After exposure to animal lungs, MoS_2_ nanosheets induced inflammatory cytokine (IL-8, TNF-α, and IL-1β) production in bronchoalveolar lavage fluids^9^. In contrary of these toxicity reports, McManus et al. found that water-based MoS_2_ and WS_2_ nanosheets induced little cytotoxicity in A549 and HaCat cells^10^. The differences of material source, physicochemical properties of TMDs, exposure time, doses and routes may be responsible for the conflict reports. Wang et al. used same cell lines (THP-1 and BEAS-2B) to us for cytotoxicity assessments and found limited cell viability changes at 24 h incubation with 0–50 μg/mL MoS_2_ nanosheets^9^, whereas we merely observed significant cytotoxicity at 48 h incubation with 50–200 μg/mL MoS_2_ nanosheets, indicating that the exposure time and doses of TMDs may greatly affect their cytotoxicity. In terms of the impacts of exposure routes, Mei et al. study showed that intravenous injected MoS_2_ elicited more toxicity than intraperitoneal and intragastric administration^11^. Under similar doses, same exposure time, and route, we and Wang et al. discovered similar pulmonary inflammation effects for MoS_2_ nanosheets^9^. Besides, consistent with our nano-SAR findings, the surface chemistry of TMDs may play a major role in their toxicities, evidenced by the increased biocompatibility of MoS_2_ nanosheets functionalized by Pluronic 127^47^, Pluronic 87^9^ and PEG molecules^48^.

According to lifecycle analysis of nanoproducts^11^, fine particles may be released into environments during the fabrication, transportation, consumption and recycling of nanoproducts. The particulates have shown high inhalation exposure risk and are capable of passing through blood-air barrier to induce severe pulmonary diseases^43^, such as inflammation, fibrosis, pneumoconiosis or chronic obstructive pulmonary disease. Since TMDs are popularly used as lubricants in industries and daily life, the fine particulates of TMDs in aerosols have high risks of inhalation exposure. The OSHA permissible exposure thresholds for soluble and insoluble molybdenum materials in workroom air are set at 5 and 15 mg/m^3^, respectively^49^. Based on a calculation approach being used at NIOSH^50^, we could estimate that a worker exposure to 15 mg/m^3^ TMDs for 8 h/day over a five-month time period over a 5-year working time could lead to a lung burden of 1.875 mg/m^2^. Considering that a bolus exposure of 1 mg/Kg in the mouse is equivalent to 0.5 mg/m^2^ of nanoparticle exposure in mouse lungs, oropharyngeal aspiration of 0–3.6 mg/Kg TMDs in mice is comparable to the real exposure levels of TMDs in workroom air. Assuming a homogeneous distribution of TMD in tissue culture dish and 10 μm thickness of cell layer^49^, the corresponding in vitro TMD dose would be 0–187.5 μg/mL. Overall, the in vitro (0–200 μg/mL) and in vivo (1 mg/Kg) doses used in this study is relevant to the possible occupational exposure in real scenarios. MoS_2_ and WS_2_ induced ferroptosis in alveolar macrophages and acute inflammation in lung tissues after 40 h exposure. The identified nano-SAR in cells could be well-validated in mouse lungs, and facilitate the safe design of TMD derivatives. Since U.S. FDA and Organisation for Economic Co-operation and Development have released strict guidance for the safety evaluation of nanoproducts^50^, the nano-SAR based safe design approach may greatly improve the sustainable development of TMD-based nanotechnology.

In summary, our study defined a TMD-induced ferroptosis effect in mammals. The ferroptosis pathway involved endocytosis of TMD nanosheets into lysosomes, GPX4 reduction, ferrous ion release, ROS generation, lipid peroxidation and cell death. Surface vacancies on WS_2_ and MoS_2_ nanosheets were responsible for ferroptosis effects. Na_2_S and methanol treatments were able to passivate particle surfaces, prevent lysosomal release of Fe^2+^ into cytoplasm and therefore protect cells. These in vitro findings could be validated in animal lungs by oropharyngeal aspiration of TMD suspensions. WS_2_ and MoS_2_ nanosheets induced ferroptosis in alveolar macrophages, pro-inflammatory cytokine production in BALF and focal inflammation around airways, whereas surface passivation could dramatically reduce these hazard effects. The discovery of TMD-induced ferroptosis as well as related nano-SARs may greatly facilitate the safe design of TMD nanoproducts as lubricants.

## Methods

### Source of materials

Bulk 2D nanomaterials were purchased from Alfa Aesar (Ward Hill, MA, USA); lysosome isolation kit, ferostatin-1, Pluronic F68, iodixanol (60% w/v), cytochalasin D, NAC, FITC labeled bovine serum albumin (FITC-BSA) and DOX were purchased from Sigma-Aldrich (St. Louis, Mo, USA); MTS and ATP assay kits were purchased from Promega (Madison, WI, USA); LysoTracker™ Red DND-99, Hoechst 33342, H_2_DCF-DA, lipid peroxidation kit, LIVE/DEAD staining kit and wheat germ agglutinin (WGA) were purchased from Thermo Fisher Scientific (Grand Island, NY, USA); FeRhoNox^TM^-1 was purchased from Goryo Chemical (Chuo-ku, Tokyo, Japan); Na_2_S was purchased from Aladdin (Shanghai, China); ELISA kits for detection of IL-1β and TNF-α were purchased from BD biosciences (San Jose, CA, USA); LIX ELISA kit was purchased from R&D (Minneapolis, MN, USA); Anti-GPX4, CHX, Z-VAD-FMK and Nec-1 were purchased from Abcam (Cambridge, MA, USA); DFP and DFX were purchased from Cayman Chemical (Ann Arbor, MI, USA); Quick-Diff staining kit was purchased from Yeasen Biotechnology (Shanghai, China); 1,2-distearoyl-sn-glycero-3-phosphocholine (DSPC) was purchased from Avanti Polar Lipids (Alabaster, Alabama, USA); RIPA lysis buffer was purchased from Beyotime (Shanghai, China); ECL hypersensitive chemiluminescence solution was purchased from BOSTER (Wuhan, China); SPA, pMD and pLKO (wide-type Trfc control) plasmids, and shRNA transfectants of target genes (Trfc1, Trfc2) were supplied by Dr. Sijin Liu at the Research Center for Eco-Environmental Sciences, Chinese Academy of Sciences, Beijing, China.

### Synthesis of 2D nanomaterials

2D nanomaterials were prepared by a reported method with a little modification^51^. In detail, 5 g of each bulk material was added into a glass flask with 350 mL of 2% w/v Pluronic F68 (PF-68) aqueous solution and dispersed by an ultrasonication equipped with a 0.125-inch probe at an operation power of 60 W (30 s on, 5 s off) for 6 h in an ice water bath. The resulted TMD suspensions (32 mL) were added into 6 mL iodixanol (60% w/v), then the deposits were consecutively collected by centrifuged (Allegra 64 R, Beckman, USA) at 2000, 4000 and 8000 rpm for 30 min, and denoted as TMD-1, TMD-2 and TMD-3 (Table 1). The supernatants (25 mL) acquired at 8000 rpm were transferred into polycarbonate tubes (Catalog: 355618, Beckman, USA) and collected the pellets by ultracentrifugation (Optima L-100XP, Beckman, USA) equipped with a 70 Ti rotor at 25,000 rpm (MoSe_2_-4 and WSe_2_-4) 50,000 rpm (MoS_2_-4, WS_2_-4, MoSe_2_-5, and WSe_2_-5) for 15 min. MoS_2_-5 (me-MoS_2_) and WS_2_-5 (ss-WS_2_) were acquired by centrifugation after 24 h reaction of 0.5 mg/mL MoS_2_-4 and WS_2_-4 in methanol and 30 mM NaS_2,_ respectively at 37 °C in a shaking incubator (100 rpm). FITC-labeled WS_2_ and MoS_2_ nanosheets were synthesized by a carboxyl-amine coupling reaction^6^. In detail, EDC (5 mg), NHS (10 mg) and 5 mg/mL FITC-BSA solution (1 mL) were sequentially added into an 5 mL Eppendorf tube containing 1 mL WS_2_ or MoS_2_ suspensions (5 mg/mL). The mixtures were sufficiently dispersed in a water bath sonication for 10 min, followed by 2 h reaction at room temperature under magnetic stirring. The resulting FITC-BSA labeled WS_2_ and MoS_2_ pellets were collected by centrifugation at 18,000 rpm for 5 min. After washing by DI water for five times, all these TMD nanomaterials were collected by centrifugation, quantified by ICP-OES to form 5 mg/mL stock solutions and stored in brown glass vials at 4 °C. These materials were characterized by AFM (Dimension Icon, Germany), confocal Raman microscopy (HR-800, JY, France), XPS (EXCALAB 250 XI, Thermo Fisher Scientific, USA), EPR (MS-5000, Magnettech, Germany), HR-TEM (Titan Cubed, Thermo Fisher Scientific, USA) and dynamic light scattering (Zetasizer Nano ZS90, Malvern).

### Preparation of TMD suspensions

TMD stock solutions were dispersed in a sonication water bath at 32 W for 10 min before adding into RPMI 1640 medium or BEGM. Desired amounts of TMD stock solutions were diluted in culture media and fully suspended by probe sonication at 20 W for 20 s.

### Cell transfection

PLKO (24 μg, 14 μL), shRNA (14.5 μg Trfc1, 13.3 μg Trfc2, 14 μL), SPA (17.9 μg, 14.5 μL), pMD (6.0 μg, 5.3 μL) and CaCl_2_ (2 M, 187.5 μL) solutions were sequentially added into a 2 mL Eppendorf tube with 1.5 mL DI H_2_O as DNA components. Aliquot of 1.5 mL N,N-bis(2-hydroxyethyl)-2-aminoethanesulfonic acid (BES) buffer was added into a 15 mL tube. Meanwhile, CaCl_2_ solution and DNA components were uniformly mixed with BES buffer and incubated for 15 min at room temperature. The resulting solution was added into 293 T cells cultured till 50% confluency in Dulbecco’s modified eagle medium (DMEM) medium (Gibco, USA) in a 75 cm^2^ flask. The post-transfection viral supernatants were collected twice at the 24 h and 48 h. Then the viral solution was acquired by centrifugation of collected supernatants at 3000 g for 15 min through a filter (0.45 μm pore size) and concentrated to 2 mL. Ultimately, aliquots of 20 μL viral solution were added into 10 mL BEGM or c-RPMI 1640 media to incubate with BEAS-2B and THP-1 cells with 50% confluency at 37 °C, respectively. After 24 h, the supernatants were replaced with complete media. The stable clones with GFP green fluorescence were selected in puromycin (10 μg/mL).

### MTS and ATP assays

BEAS-2B and THP-1 cells were cultured in RPMI 1640 medium supplemented with 10% fetal bovine serum (Gemini, Woodland, USA) and BEGM (Lonza, Walkersville, USA), respectively. While aliquots of 100 μL of THP-1 cell suspensions containing 1 μg/mL PMA were added in 96-well plates at 3 × 10^4^ cells/well, BEAS-2B cell suspensions were seeded in plates at 2 × 10^4^ cells/well. After overnight incubation, the cell media in 96-well plates were removed and replaced by aliquots of 100 μL TMD suspensions at 0, 25, 50, 100, and 200 μg/mL. After 6, 12, 24, and 48 h culture at 37 °C, the supernatants in each well were replaced by 120 μL of MTS working solution (5 mg/mL) in phenol red free media. After 2 h incubation, the plates were centrifuged at 3000 rpm for 5 min (5810 R, eppendorf, Germany) to collect the supernatants, which were transferred into 96-well plates (100 μL/well) to read OD_490_ values by a microplate reader (BioTek, Synergy neo, USA). Meanwhile, the residual cells were lyzed by ATP assay solutions. After 10 min incubation in a mini shaker (Kylin-Bell, QB-8001, China), the luminescence signals were recorded.

### Confocal microscopy imaging

The fluorphore labeled cells were observed using a confocal laser scanning microscope (FV 1200, Olympus, Japan) by a 10× or 60× oil immersion objective lens. BEAS-2B cells and PMA-primed THP-1 cells were seeded in eight-well chambers (Catalog: 155411, Lab-Tek, USA) at 1.6 × 10^5^ and 2 × 10^5^ cells/well, respectively, and cultured at 37 °C for 16 h before further treatments.

To detect lysosomal release of Fe^2+^ in BEAS-2B cells, the supernatants in each well of chambers were removed and replaced by 200 μL of nanosheet suspensions (100 μg/mL) and Fe(NH_4_)_2_(SO_4_)_2_ (100 μM) in BEGM. For alveolar macrophages, mice exposed to PBS, quartz (5 mg/Kg) and TMD (1 mg/Kg) suspensions by oropharyngeal aspiration for 40 h were sacrificed to collect cells from BALF by centrifugation (Allegra 64 R, Beckman-Coulter, USA) at 5000 rpm for 10 min. The cell suspensions in DMEM were seeded in eight-well chambers at 1 × 10^5^ cells/well. Followed by 12 h incubations, both BEAS-2B and alveolar macrophages were washed by PBS and stained by 10 μg/mL Hoechst 33342, 10 μg/mL FeRhoNox-1, and 20 μg/mL WGA in media for 1 h at 37 °C. Then, the cells were washed thrice by PBS for microscopy imaging at excitation wavelengths of 405, 488, and 543 nm.

H_2_DCF-DA staining kit was used to visualize ROS generation in cells. DFP (2 mM) and DFX (0.4 mM) were used as Fe^2+^ chelators to treat BEAS-2B cells for 2 h. Then the chelator-treated and untreated cells were exposed to 100 μg/mL ZnO, WS_2_, and MoS_2_ for 12 h incubation at 37 °C. Followed by thrice PBS washing, the cells were incubated with 10 μg/mL Hoechst 33342 and 10 μg/mL H_2_DCF-DA in dark for 30 min at 37 °C. The fluorescence of DCF was examined at excitation wavelength of 488 nm.

To assess lipid peroxidation, BEAS-2B cells were exposed to 100 μg/mL WS_2_ and MoS_2_ and incubated for 12 h at 37 °C. Cells treated with 10 μM CH for 2 h were also included as a positive control. The treated cells were washed twice by PBS and stained by Image-iT lipid peroxidation kit according to the manufacturer’s protocol. After 1 h incubation at 37 °C, the stained cells were sufficiently washed by PBS for microscopy imaging at 510 and 590 nm emission wavelengths to visualize the oxidized and non-oxidized substrates, respectively.

To examine cell viability by LIVE/DEAD staining, BEAS-2B cells were exposed to 100 μg/mL WS_2_, MoS_2_, WSe_2_, MoSe_2_, and BN in BEGM and incubated for 12 h at 37 °C. The treated cells were washed twice by PBS and incubated with 10 μg/mL LIVE/DEAD viability assay kit for 20 min at 37 °C before confocal imaging at 517 and 617 nm.

### Hemolysis assay

Blood from C57BL/6 mouse were suspended in 20 mL pre-cold PBS at 4 °C and sufficiently washed and centrifuged at 3000 rpm until the supernatant is visually clear. The cell pellets were resuspended in PBS at a density of 1 × 10^8^ cells/mL. Aliquots of cell suspensions (490 μL) were added into 1.5 mL eppendorf tubes containing 10 μL nanoparticle suspensions (5 mg/mL). The red blood cells exposed to 10 μL of DI H_2_O and 0.1% Triton X-100 were included as negative and positive controls, respectively. After 2 h incubation at 37 °C, the supernatants were collected by centrifugation at 20,000 rpm for 10 min and transferred into 96-well plates (100 μL/well) to detect the absorbance of released hemoglobin by a microplate reader (BioTek, Synergy neo, USA) at 541 nm.

### Using Nile Red labeled liposomes to simulate TMD-membrane interactions

Liposomes were prepared by thin-film ultrasonication method^52^. In detail, 1 mL of 10 mg/mL DSPC was dispersed in 6 mL chloroform in a round bottom flask. The lipid film was prepared by rotary evaporation at 60 °C. Then the lipid film was hydrated in 10 mL DI H_2_O by water-bath sonication (60 W) for 30 min. The resulted liposomes (1 mg/mL) were collected by ultrasonication at 20,000 rpm for 5 min, and resuspended in 1 mL 8 μg/mL Nile red for 2 h incubation at room temperature. After that, the labeled liposomes were washed thrice by centrifugation at 20,000 rpm for 5 min and suspended in 1 mL DI H_2_O. Nanosheets (200 μg/mL) were incubated with 1 mL labeled liposomes or DI H_2_O (blank) at 37 °C for 4 h. The suspensions were centrifuged at 10,000 rpm for 10 min to collect nanosheet pellets, followed by twice washing in DI H_2_O. The TMD pellets were subsequently dispersed in 100 μL DI H_2_O to examine the fluorescence of labeled lipids at excitation wavelength of 543 nm.

### Abiotic DCF assay

H_2_DCF-DA (50 μg) was hydrolyzed by incubation with 280 μL of 0.01 M NaOH in dark at room temperature for 30 min. The resulted H_2_DCF solution was diluted with 1720 μL PSF to form working solutions (25 μg/mL). Aliquots of 5 μL nanosheet suspensions at 5 mg/mL were added into each well in a 96-multiwell black plate (Corning, USA), and subsequently mixed with 95 μL H_2_DCF working solution. After 2 h incubation at room temperature, the fluorescence of each well was recorded at 490 nm excitation and 520 nm emission wavelengths by a microplate reader.

### Western blotting

BEAS-2B cells, alveolar macrophages and lung tissues treated with pharmacological agents or TMDs were lysed in a RIPA lysis buffer. The protein concentrations were determined and adjusted by Bradford assay. The cell lysates were separated on a 12% SDS-polyacrylamide gel (Beyotime, Shanghai, China) at 120 V and transferred to a nitrocellulose membrane at 300 mA. The membranes were blocked with 5% milk (Biofroxx, Einhausen, Germany) in 0.1% PBS/Tween for 2 h at room temperature, and then incubated with primary antibody (1/1000 in blocking buffer) for 16 h at 4 °C. After five-time washing with PBS/Tween and incubation with HRP-conjugated secondary antibody (1/1000 in PBS) for 1 h at room temperature, membranes were sufficiently washed by PBS/Tween 20 at a 5 min interval for five times. A freshly prepared ECL hypersensitive chemiluminescence solution was used to develop the membranes, which were imaged by multi-color fluorescence chemiluminescence imaging analysis system (FluorChem M, Alpha, USA). The unprocessed blot scans were added in Supplementary Fig. 13.

### Animal experiments

Eight-week-old female C57BL/6 mice, which were obtained from Nanjing Peng Sheng Biological Technology (Nanjing, Jiangsu, China), were housed in Laboratory Animal Center of Soochow University. Our animal protocols were approved by the Committee of Animal Research and Ethics in Soochow University. Mice were exposed to 0.5, 1, 2, 4 mg/Kg WS_2_, or 1 mg/Kg MoS_2_, ss-WS_2_, me-MoS_2_, and MoSe_2_ by oropharyngeal aspiration. In detail, 2D nanosheets suspended in PBS were instilled at the back of the tongue with 50 µL suspensions in anesthetized animals (*n* = 6). Animals instilled 50 µL PBS and quartz solutions (5 mg/Kg) were used as vehicle and positive controls, respectively. After 40 h exposure, mice were sacrificed to acquire BALF. While the immune cells in BALF were collected by centrifugation at 8000 rpm for cell counting, the supernatants were used for detection of LIX, IL-1β, and TNF-α by ELISA. The lung tissues were fixed for H&E staining to examine pathology changes.

### Statistics

Data analysis was performed by Student *t*-test. The difference is regarded as statistical significance if *p* < 0.05. Data are reported as the mean ± standard deviation from at least three replicates.

### Reporting summary

Further information on research design is available in the Nature Research Reporting Summary linked to this article.

## Supplementary information

Supplementary Information

Peer Review File

Supplementary Movie 1

Supplementary Movie 2

Supplementary Movie 3

Reporting Summary

## Data Availability

The datasets generated and/or analysed during the current study are available in the Harvard Dataverse repository, https://dataverse.harvard.edu/dataset.xhtml?persistentId=doi:10.7910/DVN/OZLXPB.
