# Supplementary material for: Vacancies on 2D transition metal dichalcogenides elicit ferroptotic cell death
Source: Nat Commun. 2020 Jul 13;11:3484. doi: 10.1038/s41467-020-17300-7 (PMC7359333; doi:10.1038/s41467-020-17300-7)
Supplement: Supplementary file 1 — Supplementary Information [file 41467_2020_17300_MOESM1_ESM.pdf]

Supplementary Materials

Vacancies on 2D Transition Metal dichalcogenides Elicit Ferroptotic Cell Death

Shujuan Xu[1], Huizhen Zheng[1], Ronglin Ma[1], Di Wu[1], Yanxia Pan[1], Chunyang Yin[2], Meng Gao[1], Weili Wang[1], Wei Li[1], Sijin Liu[2], Zhifang Chai[1], Ruibin Li[1*]

[1] State Key Laboratory of Radiation Medicine and Protection, School for Radiological and Interdisciplinary Sciences (RAD-X), Collaborative Innovation Center of Radiological Medicine of Jiangsu Higher Education Institutions, Soochow University, Suzhou 215123, Jiangsu China

[2] State Key Laboratory of Environmental Chemistry and Ecotoxicology, Research Center for Eco-Environmental Sciences, Chinese Academy of Sciences, 18 Shuangqing Road, Beijing 100085, China

[*]Corresponding author: Dr. Ruibin Li

Email: liruibin@suda.edu.cn

Supplementary Table 1 Hydrodynamic size and ζ-potential of 2D materials in media

2D nanomaterials	ζ-potential (mV) (water)	Hydrodynamic diameter (nm)		
		water	RPMI 1640	BEGM
WS_2	-6.04 ± 0.26	94 ± 2	104 ± 26	120 ± 15
MoS_2	-18.55 ± 0.32	91 ± 1	100 ± 2	94 ± 2
WSe_2	-24.18 ± 0.27	94 ± 1	97 ± 1	94 ± 1
$MoSe_2$	-18.70 ± 0.41	123 ± 2	124 ± 4	129 ± 2
BN	-25.35 ± 0.54	126 ± 2	132 ± 2	131 ± 2

Data are presented as mean values ± SD. Replicate numbers: ζ-potential (n=10) and hydrodynamic diameter (n=10).

A)

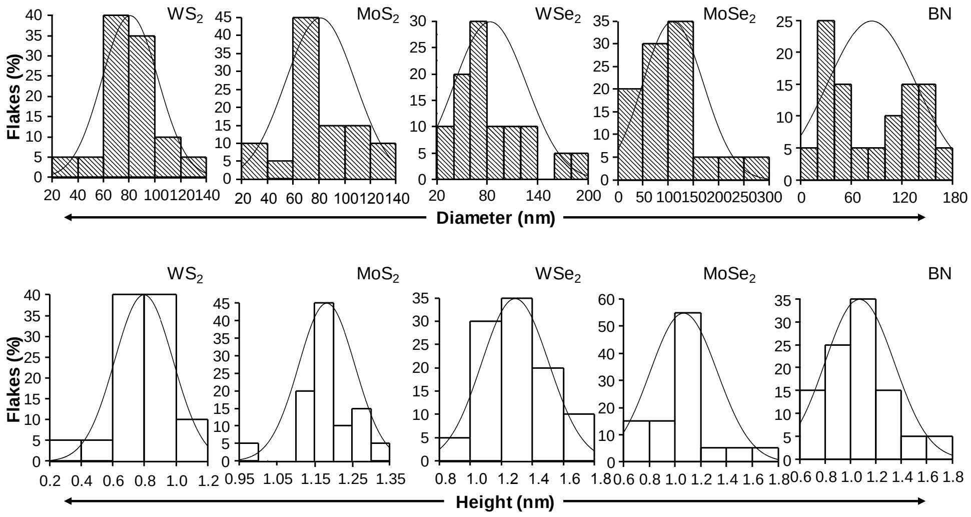

B)

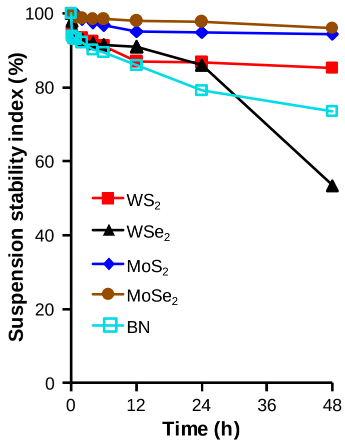

Supplementary Figure 1 Characterization of particle size and stability

A) AFM histograms of 2D TMDs and **B)** suspension stability of 2D TMDs in RPMI 1640 media (n=3 independent experiments). The flake diameter and height of 2D TMDs were calculated from AFM images by Nano Measurer and Nanoscope. Stability test was performed by examining the absorbance of TMD suspensions (100 μg/mL) on a UV-Vis spectrophotometer (UV-3600, SHIMADZU, Japan).

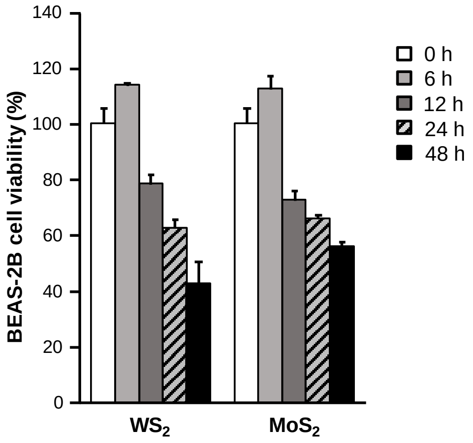

Supplementary Figure 2 Cytotoxicity tests at different time points

BEAS-2B cells were exposed to 200 μg/mL WS_2 and MoS_2 nanosheets for 6 h, 12 h, 24 h and 48 h. Cell viability was examined by ATP assay (n=3 biologically independent cell samples). Data are presented as mean values ± SD.

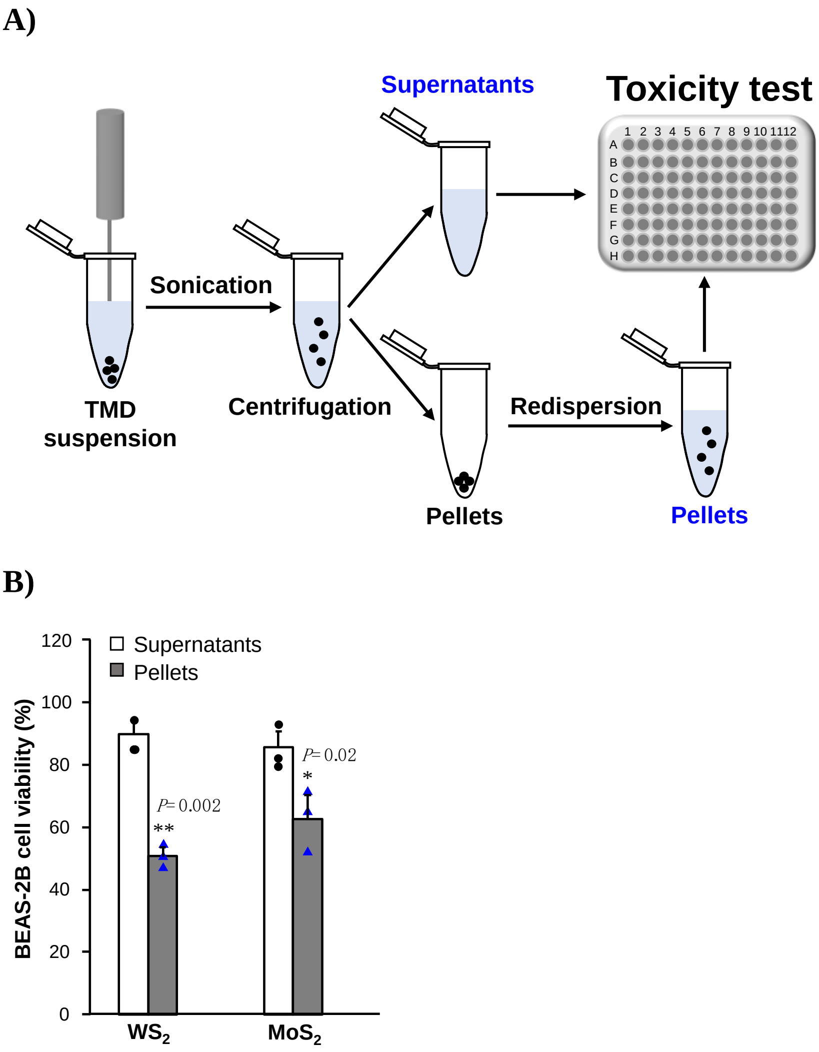

Supplementary Figure 3 Impacts of TMD dissolution on cytotoxicity

A) Schematics to separate dissolved ions from TMD pellets and **B)** comparison of cytotoxicity in BEAS-2B cells exposed to TMD supernatants and pellets. WS_2 or MoS_2 (200 μg/mL) dispersed in BEGM were centrifuged to separate the supernatants and pellets. BEAS-2B cells were exposed to supernatants and pellets for 48 h to examine cell viability (n=3 biologically independent cell samples). Data are presented as mean values ± SD. * $p < 0.05$, ** $p < 0.01$ compared to TMD pellet treatments by two-tailored Student T-test.

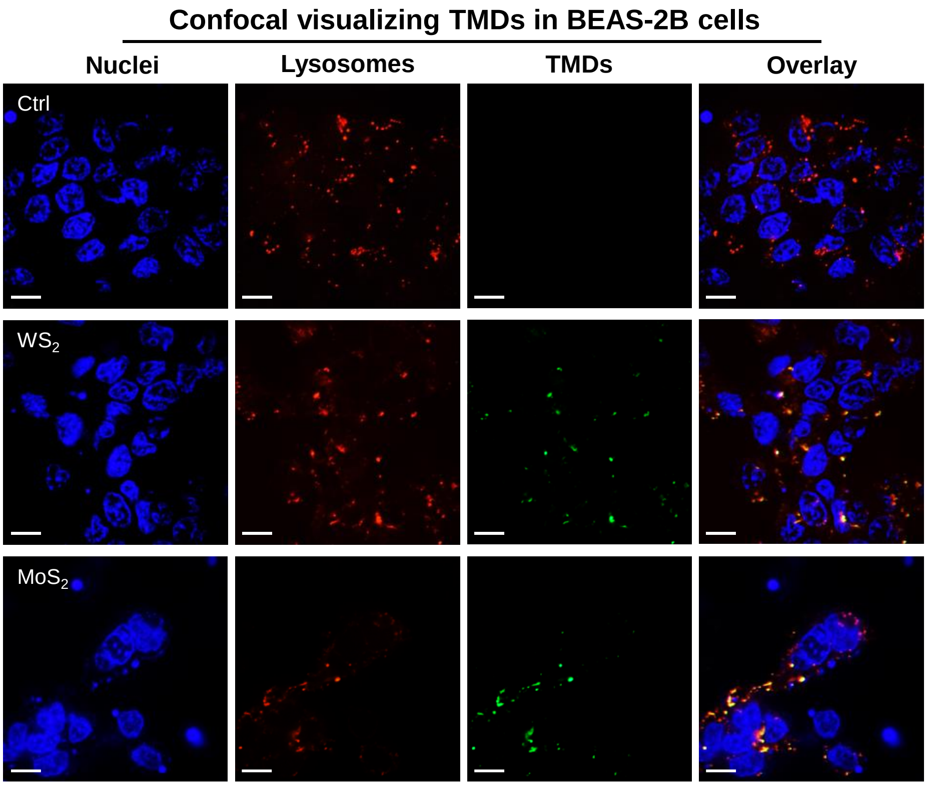

Supplementary Figure 4 Representative confocal images of WS_2 and MoS_2 distributions in cells

WS_2 and MoS_2 were labeled by FITC-BSA (green). BEAS-2B cells treated by 100 μg/mL WS_2 and MoS_2 for 24 h were stained by Hoechst 33342 (blue) and lysosomal tracker (red) to visualize the nuclei and lysosome in cells, respectively (scale bar, 10 μm). Shown are the representative images from three independent experiments.

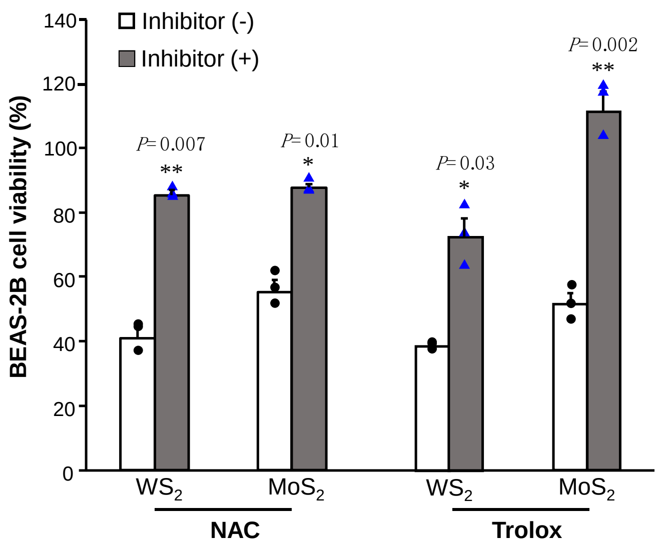

Supplementary Figure 5 Impacts of ROS inhibitor on WS_2 and MoS_2 induced cytotoxicity

BEAS-2B cells pretreated with 5 mM NAC for 2 h or 50 μM Trolox for 1 h were exposed to 200 μg/mL WS_2/MoS_2. After 48 h, cell viability was examined by ATP or MTS assay (n=3 biologically independent cell samples). Data are presented as mean values ± SD. * $p < 0.05$, ** $p < 0.01$ compared to cells without NAC or Trolox treatment by two-tailored Student T-test.

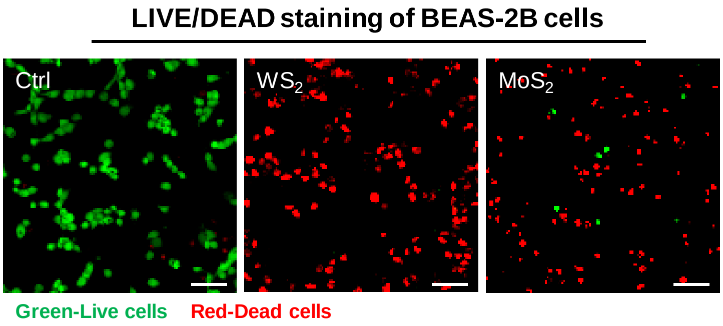

Supplementary Figure 6 Representative images of WS_2/MoS_2 induced cell deaths by LIVE/DEAD staining

BEAS-2B cells exposed to 100 μg/mL WS_2 and MoS_2 for 12 h were acquired to stain with LIVE/DEAD staining kit. After 20 min, the stained cells were visualized by a confocal laser scanning microscope (FV 1200, Olympus, Japan) (scale bar: 100 μm). Shown are the representative images from three cell samples.

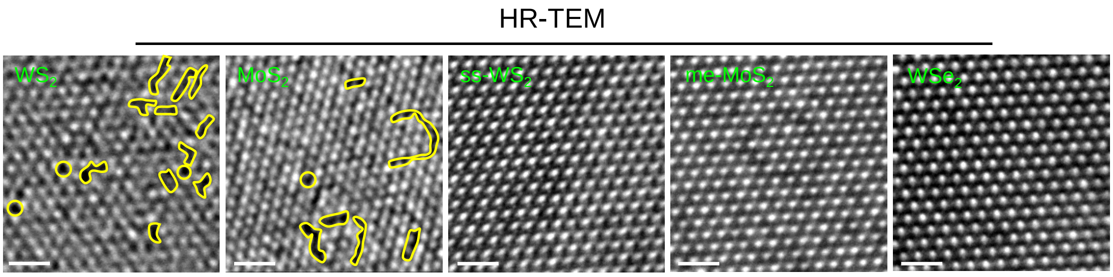

Supplementary Figure 7 Representative HR-TEM images of vacancies on pristine and modified TMD surfaces

WS_2, MoS_2, ss-WS_2, me-MoS_2 and $MoSe_2$ nanosheets were dropped on ultra-thin carbon film to visualize atom arrangement by HR-TEM (Titan Cubed, Thermo Fisher Scientific) under an electron beam flux of ~50 A/cm^2 at 80 kV (n=15 independent views). Irregular-shaped yellow circles indicate surface vacancies. Shown are the representative images from three experiments. The scale bar is 1 nm.

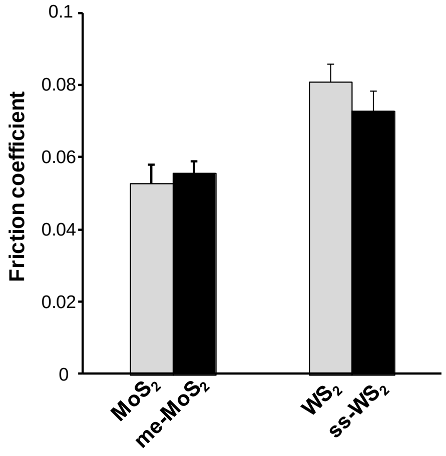

Supplementary Figure 8 Friction coefficients of pristine and functionalized MoS_2 and WS_2

Lubricant (100 mg) was added to the contact area on an Optimal-SRV-IV tribometer (SRV load, 100 N; temperature, 25 ℃; stroke, 1 mm; frequency, 25 Hz). The friction coefficient was recorded automatically *via* a computer connected to the SRV tester (n=3 independent experiments). Data are presented as mean values ± SD.

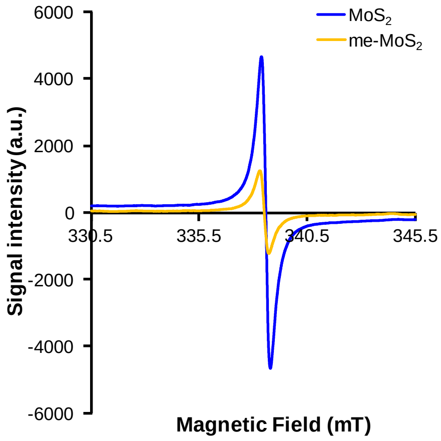

Supplementary Figure 9 EPR spectra of pristine and safer designed MoS_2

The power samples (10 mg) of MoS_2 and Me-MoS_2 were subjected to EPR (MS-5000， Magnettech， Germany) at a g value of 2.003133.

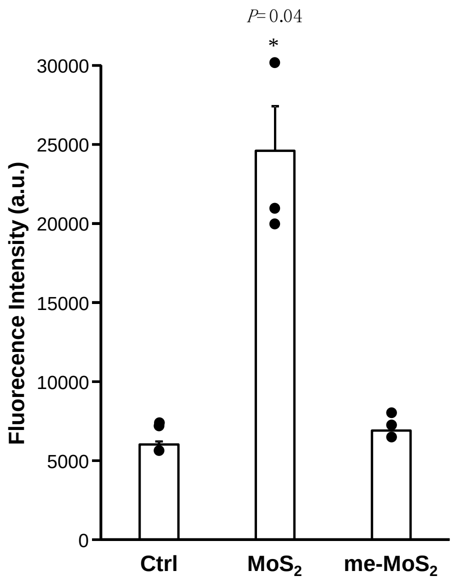

Supplementary Figure 10 Oxidative damages of lysosomes by MoS_2

Lysosomes extracted from BEAS-2B cells were incubated with 200 μg/mL MoS_2 for 2 h. After centrifugation at 16000 rpm/min for 10 min, the supernatants were incubated with lipid peroxidation assay kit for 2 h to examine the oxidation damages in MoS_2 treated lysosomes (n=3 independent experiments). Data are presented as mean values ± SD. *p < 0.05 compared to Ctrl by two-tailored student T-test.

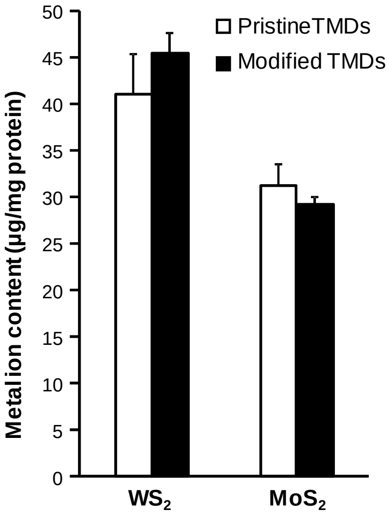

Supplementary Figure 11 Impacts of surface passivation on cellular uptake

BEAS-2B cells were exposed to WS_2, MoS_2, ss-WS_2 and me-MoS_2 at the concentration of 100 μg/mL for 24 h. Then the cells were collected, sufficiently washed and digested for ICP-OES detection (n=3 independent samples). Data are presented as mean values ± SD.

A B

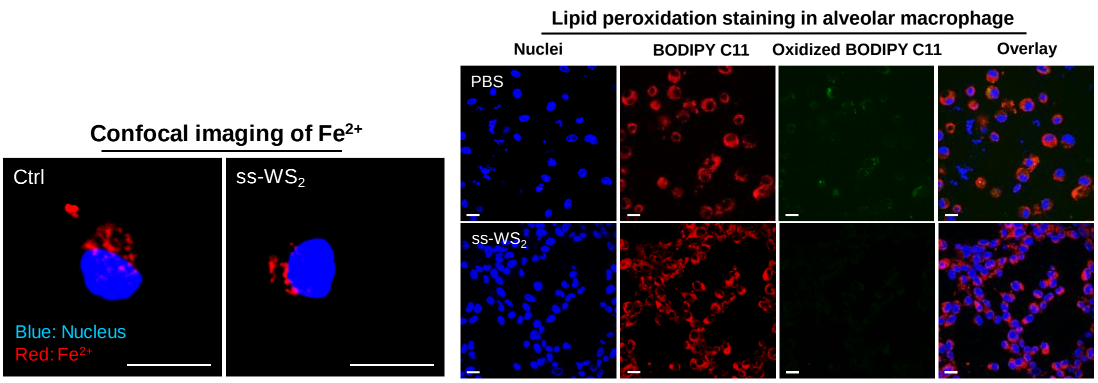

Supplementary Figure 12 Impacts of surface passivation on WS_2 induced ferroptosis in alveolar macrophages

Representative confocal images of **A)** Fe^{2+} and **B)** lipid peroxidation in alveolar macrophages from 10 independent views. Animals received oropharyngeal aspiration of 1 mg/Kg ss-WS_2 were sacrificed at 40 h to collect macrophages from BALF (n=3 mice). The alveolar macrophages were seeded in eight-well chamber and stained by FeRhoNox-1 to visualize Fe^{2+}, or Image-iT lipid peroxidation staining kit to observe nuclei (blue), reduced substrate (red) and oxidized substrate (green) by confocal microscopy (scale bar: 10 μm).

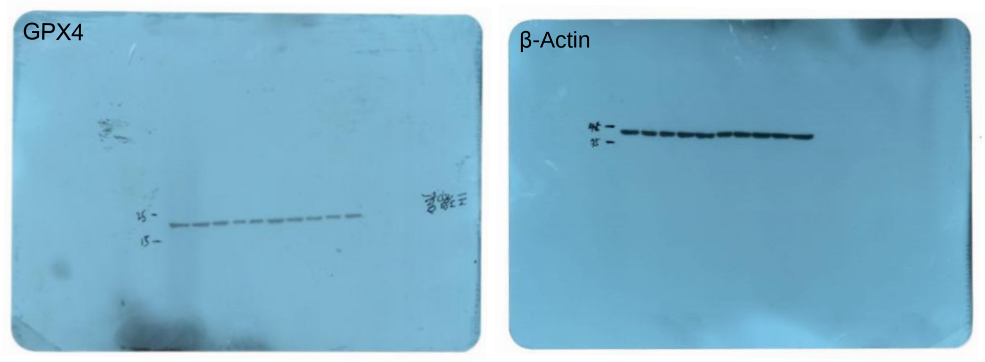

Supplementary Figure 13 Representative unprocessed western blotting scans
