# Supplementary material for: Vacancies on 2D transition metal dichalcogenides elicit ferroptotic cell death
Source: Nat Commun. 2020 Jul 13;11:3484. doi: 10.1038/s41467-020-17300-7 (PMC7359333; doi:10.1038/s41467-020-17300-7)
Supplement: Supplementary file 6 — Reporting Summary [file 41467_2020_17300_MOESM6_ESM.pdf]

nature research

Corresponding author(s): Ruibin Li

Last updated by author(s): June 3, 2020

Reporting Summary

Nature Research wishes to improve the reproducibility of the work that we publish. This form provides structure for consistency and transparency in reporting. For further information on Nature Research policies, see Authors & Referees and the Editorial Policy Checklist.

Statistics

For all statistical analyses, confirm that the following items are present in the figure legend, table legend, main text, or Methods section.

n/a	Confirmed	
☐	☒	The exact sample size (*n*) for each experimental group/condition, given as a discrete number and unit of measurement
☐	☒	A statement on whether measurements were taken from distinct samples or whether the same sample was measured repeatedly
☐	☒	The statistical test(s) used AND whether they are one- or two-sided<br>*Only common tests should be described solely by name; describe more complex techniques in the Methods section.*
☒	☐	A description of all covariates tested
☒	☐	A description of any assumptions or corrections, such as tests of normality and adjustment for multiple comparisons
☐	☒	A full description of the statistical parameters including central tendency (e.g. means) or other basic estimates (e.g. regression coefficient) AND variation (e.g. standard deviation) or associated estimates of uncertainty (e.g. confidence intervals)
☐	☒	For null hypothesis testing, the test statistic (e.g. *F*, *t*, *r*) with confidence intervals, effect sizes, degrees of freedom and *P* value noted<br>*Give P values as exact values whenever suitable.*
☒	☐	For Bayesian analysis, information on the choice of priors and Markov chain Monte Carlo settings
☒	☐	For hierarchical and complex designs, identification of the appropriate level for tests and full reporting of outcomes
☐	☒	Estimates of effect sizes (e.g. Cohen's *d*, Pearson's *r*), indicating how they were calculated

Our web collection on statistics for biologists contains articles on many of the points above.

Software and code

Policy information about availability of computer code

Data collection	No Software was used for data collection.
Data analysis	No Software was used for data analysis.

For manuscripts utilizing custom algorithms or software that are central to the research but not yet described in published literature, software must be made available to editors/reviewers. We strongly encourage code deposition in a community repository (e.g. GitHub). See the Nature Research guidelines for submitting code & software for further information.

Data

Policy information about availability of data

All manuscripts must include a data availability statement. This statement should provide the following information, where applicable:

- Accession codes, unique identifiers, or web links for publicly available datasets
- A list of figures that have associated raw data
- A description of any restrictions on data availability

The datasets generated and/or analysed during the current study are available in the Harvard Dataverse repository, https://dataverse.harvard.edu/dataset.xhtml?persistentId=doi:10.7910/DVN/OZLXPB.

Field-specific reporting

Please select the one below that is the best fit for your research. If you are not sure, read the appropriate sections before making your selection.

☒ Life sciences ☐ Behavioural & social sciences ☐ Ecological, evolutionary & environmental sciences

Life sciences study design

All studies must disclose on these points even when the disclosure is negative.

Sample size	Mead's resource equation was used to determine animal numbers. The number of cell samples were determined based on the preliminary test results as well as the minimal requirements in Student T-test.
Data exclusions	No data were excluded in the study.
Replication	Attempts at replication were performed in each experiment until three consecutive successful attempts were made. Following are the details:<br>Nanoparticle synthesis and characterization experiments: all attempts are successful (Figure 1,Supplementary Figure 1 and supplementary table 1);<br>Suspension stability experiment: all attempts are successful (Supplementary Figure 1B);<br>MTS and ATP experiments: all attempts are successful (Figure 1B and S2);<br>Z-VAD and Nec-1 inhibition experiments: all attempts are successful (Figure 1C);<br>Cytochalasin D, DFP and DFX, NAC inhibition experiments: one of the four relicates are failed (Figure 2B, 3B and S5);<br>TfR-KD in THP-1 and BEAS-2B cell experiments: all attempts are successful (Figure 3C);<br>Western blotting experiments of GPX4: two of the five relicates are failed (Figure 4A);<br>Fer-1 treatment experiments:all attempts are successful (Figure 4B)<br>Confocal imaging of Fe2+ , ROS, lipid peroxidation and LIVE/DEAD experiments : all attempts are successful (Figure 3A, 3D, 3E, 5C, 6B and 6C);<br>Neutrophil counts and cytokine release experiments: all attempts are successful (Figure 7A and 7B)<br>H&E staining: one of the six relicates are failed (Figure 7C)
Randomization	All the cell and animal experiments were randomly allocated into experimental groups by throwing dice.
Blinding	Blind trials were performed in nanoparticle synthesis, some nanoparticle characterizations (EPR, AFM, DLS, UV-Vis), cell imaging, cell viability tests, western blotting, animal experiments (e.g. differential cell counting, cytokine production and H&E staining). Blinding was not possible in a few characterization experiments because the operator could easily identify the compositions by characteristic signals (Raman, TEM) or have to known the elemental composition for instrument setting (ICP-OES).

Reporting for specific materials, systems and methods

We require information from authors about some types of materials, experimental systems and methods used in many studies. Here, indicate whether each material, system or method listed is relevant to your study. If you are not sure if a list item applies to your research, read the appropriate section before selecting a response.

Materials & experimental systems

n/a	Involved in the study	
☐	☒	Antibodies
☐	☒	Eukaryotic cell lines
☒	☐	Palaeontology
☐	☒	Animals and other organisms
☒	☐	Human research participants
☒	☐	Clinical data

Methods

n/a	Involved in the study	
☒	☐	ChIP-seq
☒	☐	Flow cytometry
☒	☐	MRI-based neuroimaging

Antibodies

Antibodies used	Anti-IL-1beta (Cat#: 559623 mouse) and Anti-TNF-alpha (Cat#: 555268 mouse) in ELISA kit were purchased from BD biosciences (San Jose, CA, USA), Anti-LIX (Cat#: 890803 mouse) in ELISA kit was purchased from RD biosciences (Minneapolis, MN, USA); Anti-GPX4 (Cat#: ab125066) was purchased from Abcam (Cambridge, MA, USA).
Validation	The ELISA kit were validated in our previous publications (ACS Nano, 2014, 1771; Nature Communications, 2018, 4416), Anti-GPX4 was validated in literature reports (Science Advance, 2019, eaau7314)

Eukaryotic cell lines

Policy information about cell lines

Cell line source(s)	THP-1 cells, BEAS-2B cells and 293T cells were purchased from ATCC.
Authentication	THP-1 cells were seeded in complete RPMI medium and primed with 1 µg/mL phorbol 12-myristate acetate (PMA) overnight before use (Li et al, ACS Nano, 2013, 2352; Wang et al, Nano Letters, 2012, 3050). BEAS-2B and 293T cells were authenticated by the positive staining of SV40 T antigen.

Mycoplasma contamination	Our cell lines are negative for mycoplasma contamination.
Commonly misidentified lines (See ICLAC register)	We double checked our cell lines (THP-1 and BEAS-2B) in version 8.0 of ICLAC. No commonly misidentified cell lines were used in our study.

Animals and other organisms

Policy information about studies involving animals; ARRIVE guidelines recommended for reporting animal research

Laboratory animals	Eight-week-old male or female C57BL/6 mice, which were obtained from Nanjing Peng Sheng Biological Technology (Nanjing, Jiangsu, China), were housed in Specific Pathogen Free (SPF) level, ambient temperature of 20-26 ºC, 40%-70% humidity and 12 h dark/12 h light cycle in Laboratory Animal Center of Soochow University.
Wild animals	No wild animals were used in the study.
Field-collected samples	No field collected samples were used in the study.
Ethics oversight	Committee of Animal Research and Ethics in Soochow University

Note that full information on the approval of the study protocol must also be provided in the manuscript.
