# Supplementary material for: Vacancies on 2D transition metal dichalcogenides elicit ferroptotic cell death
Source: Nat Commun. 2020 Jul 13;11:3484. doi: 10.1038/s41467-020-17300-7 (PMC7359333; doi:10.1038/s41467-020-17300-7)
Supplement: Supplementary file 2 — Peer Review File [file 41467_2020_17300_MOESM2_ESM.docx]

Peer Review Information

**Journal:** Nature Communications

**Manuscript title:** Vacancies on 2D Transition Metal Dichalcogenides Elicit Ferroptotic Cell Death

**Corresponding author name(s):** Prof Ruibin Li

**Editorial notes:**

**none**

**Reviewer comments & decisions:**

**Reviewer comments, first version:**

Reviewer #1 (Remarks to the Author):

Xu et al explored the metal dichalcognides nanoparticles induced ferroptotic cell death in BEAS-2B cells. The role of nanoparticles on ferroptosis is importance in the field of cancer and other disease as well. The manuscript is well written and supports their conclusion. Authors need to include some additional studies to strengthen the story. The analysis of the manuscript raises the following criticisms:
1. Fig 1C- authors reported WS2 and MoS2 nanoparticles induced ferroptotic cell death, not apoptotic or necrotic death. Authors should use ferroptotic inhibitors like Ferrostatin-1 to verify ferroptotic mediated cell death.

2. Bubbling is one of the common phenotype on lipid peroxidation mediated ferroptotic cell death. It would be good to check the bubbling phenotypes in BEAS-2B cells when cell death caused by WS2 and MoS2 nanoparticles.

3. To claim WS2 and MoS2 nanoparticles induced ferroptotic cell death, authors further validate some ferrototic markers like PTGS2, GPX4 etc protein and mRNA expression.

4. Figure 6, authors measured most of the inflammatory markers in tissue. It would be ideal to check ferroptosis markers in isolated lung tissue.

5. There are numerous grammatical and typographical errors throughout the paper. Please go through overall the manuscript.


Reviewer #2 (Remarks to the Author):

The manuscript of S. Xu at all show the influence of MoS2 and WS2 on cell metabolism. The interaction of layered disulfide was reported by several authors in recent years. I don’t see sufficient significance for publication of this work in Nature Communication. In the manuscript are also not sufficient characterization of exfoliated MoS2/WS2. There is none clear evidence of vacation formation. This should be shown by HR-TEM in combination with other methods like XPS and other techniques which provide sufficient evidence of vacancies in chalcogenides sheets. I recommend to publish this work in more specialized journal.


Reviewer #3 (Remarks to the Author):

Xu et al. have studied a panel of five different 2D materials with respect to cytotoxicity and show that 2 of the materials namely WS2 and MoS2 elicit ferroptosis, an iron-dependent form of cell death in the human THP-1 and BEAS-2B cell lines in vitro). The authors have also documented a pro-inflammatory response to these materials in an animal model following pharyngeal asiration, but did not confirm ferroptosis in vivo.

The authors must carefully attend to the following major remarks:

1. The authors have overstated thee conclusion regarding the role of metal vacancies on WS2 and MoS2 surfaces (cf. the title of the manuscript). The study shows that passiviation of these materials by saturation with Na2S and methanol, respectively, results in a partal (yet significant) reduction in cytotoxicity. Hoewever, mor detailed studies are required to prove that metal vacancies are playing a key role. Furthermore, the suggestion that this amounts to "safe design" is questionable: a material can elicit more or less cytotoxicity but "safe-by-design" implies that a materal retains its useful properties while displaying a reduced toxicity; if a material is inert and no longer displays useful properties then this is passivation but not "safe design". Does the passivation also work in vivo? How stable or long-lasting is this surface saturation with methanol or Na2S? Does the passivation selectively affect ferroptosis or is it possible that other modes of programmed or non-programmed cell death also contribute to the cytotoxcity effects of the TMDs?

2. The abstract is deficient in that the authors failed to mention that they have studies five different TMDs, and they do not mention that the work was carried out usig the BEAS-2B and THP-2 cell model. The authors also need to carefluly reassess the use of the term "nanoSARs" (or stucture-activity relationship studies). One cannot extract information on structure-activity relationships of a class of materials based on the study of only 2 materials. Achieving the description of nano-SARs or informative nano-descriptors with which to predict toxcity of nanomaterials is a formidable task and one that requires studies of sufficiently large databases of nanomaterals of reasonabe diversity with respect to intrinsic properties and biological receptors; most often, computational aproaches are required (Puzyn T et al. Small. 2009;5(22):2494–2509; Liu R et al. 2011;7(8):1118-1126). The present study is NOT a nano-SAR study.

3. Introduction: This section is very poorly written and hardly provides sufficient background information for the present study. The very firts sentence is very ambiguous and seems to suggest a curious circular argument instead of simply stating that it is important to understand which propeties that are drvig the toxicity of nanomaterials; morover, the first paragrah is focused on SARs but this is not relevant for the current study). The authors also failed to provide a relevant background on previous studies of 2D materials thus giving the impression that the current study is the first to address the potential toxicity of this class of materials but this is certainly no correct. Some relevant references are reported in the discussion (page 13) but this short paragraph also fails to provide all the relevant information, and there are certainly many other reasons for the "conflict toxicity results" including not only "different sources" of the TMDs but also the use of different in vitro or in vivo models, different doses, and so on.

The authors also exaggerate the appoach taken in the present study: one cannot refer to a small panel of 5 materials as a "library" of TMDs. Studies using 81 different surface-modified MWCNts (Wu L et al. ACS Nano. 2014 Mar 25;8(3):2087-99) or 105 surface-modified gold NPs (Walkey CD et al. ACS Nano. 2014 Mar 25;8(3):2439-55) are considered as studies of "libraries" but the present study is on a small panel (n=5) of TMDs and it is NOT a nanoSAR study; no predictive information can be derived of the study of 2 different TMDs (with or without surface passivation). Again, the "safer design" of these materials is also questionable as the authors did not test the materials for their functionalit, only whether or not they caused cytotoxcity.

4. Results: the authors have used very high doses of the TMDs (up to 200 ug/mL). What is the relevance of using such high doses? For the in vivo studies, the authors applied 1 mg/mg of TMDs. What is the relation between the in vitro dose and the in vivo dose in the present study and how does the in vivo dose compare with the doses that may potentially occur in a human setting? Minor comment: the authors wite that the 2D materials were exposed to the THP-1 and BEAS-2B cells, but it is in fact the other way around. Overall, the author may want to check the manuscript for grammatical consistency (for instance, the first sentence of the main text: "nanobiology researches", and so on).

The suggestion that all materials have "similar dosimetry effects in their interactions with cells" is not supported by the data. What the authors have shown is that the materials are well dispersed i cell medium, bu the density of each material will determine the in vitro dosimetry, and there are no "dosimetry effects". To fully understand the intacellular dose, the authors need to check cellular uptake of each material.

Concerning the mode of cell death: The authors have shown that zVAD-fmk (a pan-caspase inhibitor, not an "apoptosis inhibitor") and necrostatin-1 failed to block cell death. Why did the authors not test the commonly used ferroptosis inhibior, ferrostatin-1? Why did test only NAC and not, for instance, Trolox? The results suggest a role for ROS, but mor evidence is needed to prove that this is ferroptosis. Furthermore, why did the authors not check for cell death/ferroptsis in the animal model? The authors claim that the results obtained in the animal model validated or confirmed the in vitro results but this is not correct. The in vitro studies were focused on cell death, lipid peroxidation, lysosomal involvment while the in vivo study addressed pro-inflammatory effects in the lungs of exposed mice.

The authors used cytochalasin D to show that the toxicity of the TMDs was significantly reduced and they draw the conclusion that this "suggests that lyssoe internalized WS2 and MoS2 are responsible for the downstream hazard signals" but this is also incorrect. This experiment shows that endocytosis is requird for cytotoxcity but does not give any clues regarding the lysosomes.

The results obtained with NAC (a precursor of GSH) are important. But can the authors exclude that NAC is acting as a metal chelator and not (only) as an antioxidant? The results obtained with the 2 iron chelators are also important. Again, can the authors conclusively show that these reagents bind iron (a key finding on which the entire ferroptsis story is based) as opposed to chelating other metals including the TMDs themselves?

Overall, the authors speculate too much in the results section. For instance, they claim that the experiments using PC liposomes and red blood cells (hemolysis assay) "indicate" that metal vacancies on MoS2 and WS2 "may affect the architectures of lysosomes and elicit lysosomal membrane permeabilization (LMP) by oxidative damages or phospholipid extraction". But this was not proven by the latter experiments, this is an extrapolation.

The author based a great deal of emphasis on the lysosomes and they presumed that these organelles are the source of iron in this setting, but they need to provide real evidence that the TMDs are in fact present in lysosomes, by specifc labeling of these organelles. Showing TEM images of the TMDs in cells in not sufficient evidence. As a general remark, it is not clear why the authors have placed all the results with THP-1 cells in the supplement. These results should also be presented in the main text.

The authors have used knockdown of the transferrin receptor (TfR) to "validate" the role of iron. The method for this "knockdown" is not clarified in the manuscript: which cells were used? The methods section mentions "shRNA transfectants of target genes (Trfc1, Trfc2)" (page 15) but which cells were used and where is the method description on the transfection of cells? Addiig to this, the schematic figure (Figuer 4) is completely redundant and it should be removed from the paper. This is a typical "graphical abstract" figure and does not belong in the main text (and it is unusual to "summarize" the findings in the middle of a paper. Furthermore, the figure does not shwo some of the key pieces of information, such as: the role of iron? (and the role, if any, of the transferrin receptor). As it stands, the figure only suggests that lysosomes and ROS are involved in soe way in lipid peroxidation and cell death. The "mechanism" of lysosomal damage (i.e. phospolipid extaction and/or oxidation) is also very vague and this is, in fact, a major deficiency in the current study. The authors need to present more evidence for one or both of these potential mechanisms to strengthen the story. They also need to provide more evidence that the passivation strategy actally has an impact on these mechanisms of lysosomal damage as opposed to a more general effect. For instance, surface passivation may simply lead to a reduced cellular uptake of the TMDs thereby leadig to a reduced toxicity. In other words, the passivation does not necessarily have to mping on "ferroptis" signaling in cells.

It is also noted that the experiment using TfR "knockdown" cells were conducted at a dose of 200 ug/mL (48 hours): this is very excessive and the authors need to address the dose-response, if any.

As already mentioned, the in vivo part is unsatisfactory and the results do not "duplicate the results seen in tissue culture cells". The animal study shows that th TMDs elicit pulmonary inflammation with increased expression of a couple cytokines. The authors also stated that macrophage numbers were significantly increased in BAL fluid" but they do NOT show these data (Figuer 6A shows imags of iron labeling in alveolar macrophages). Instead, what they show is that neutrophil numbers are increased (Figure 6B). Why did the authors not address whether there is any ferroptotic cell death in the lungs of mice? (and whether Trolox or other antioxidants prevents the cell death). Conversely, do TMDs elicit any cytokine release in THP-1 or BEAS-2B cells? (in particular, IL-1beta or TNF-alpha).

5. Discussion: Unfortunately, and similarly to the introduction, this section is unsatisfactory, and very brief. The authors do not provide a sufficient discussion (here or in the intoduction) of other previous studies of MoS2 or other 2D materials even though some previous stdies have shown very similar results (for instance, Wang X et al. 2015;11(38):5079-5087).
The authors, again, exaggerated the conclusion regarding "nano-SARs". One cannot claim that the present study can " determine the tiered hazard signaling pathways and nano-SARs of TMDs in mammalian cells". The present study has NOT elucicated any hazard tiers and cannot be generalized to all TMDs nor to all mammalian cells. The authors MUST focus simply on the results at hand.
The authors also claim (on page 14) that "as expected, MoS2 and WS2 induced ferroptosi in alvealar macrophages and acute inflammation in lung tissues" but this is not correct. They did NOT show ferroptosis (or any other form of cell death) in the animal model (and the results cannot be considered to be "expected"). More work is required to link the in vitro and in vivo findings. The authors did not consider the differences in possble bio-coronas under in vitro and in vivo conditions but this may also be very relevant especially if the TMDs cause oxidation of PC (a maor component of the lung surfactant fluid).

6. Figures: The figure legend of Figure 6 ("Validation of ferroptosis and nano-SAR in mouse lungs") is entirely misleading.

**Author rebuttal, first version:**

**Reviewer: 1**

Comments:

Xu et al explored the metal dichalcognides nanoparticles induced ferroptotic cell death in BEAS-2B cells. The role of nanoparticles on ferroptosis is importance in the field of cancer and other disease as well. The manuscript is well written and supports their conclusion. Authors need to include some additional studies to strengthen the story. The analysis of the manuscript raises the following criticisms:

**Response:** We thank the reviewer for thinking that our paper is well-written and support our conclusion. We have performed more experiments and added new data to address the reviewer’s concerns.

1. Fig 1C- authors reported WS2 and MoS2 nanoparticles induced ferroptotic cell death, not apoptotic or necrotic death. Authors should use ferroptotic inhibitors like Ferrostatin-1 to verify ferroptotic mediated cell death.

**Response:** Thanks for the reviewer’s suggestion. We examined the effects of ferrostatin-1 (Fer-1) in BEAS-2B and THP-1 cells. As shown in Figure R1, Fer-1 treatment could significantly ameliorate cytotoxicity induced by WS2 and MoS2 in two cell lines. These results have been added in revised manuscript as Figure 4A, and described in line 197-200, as “To further confirm the ferroptosis mechanism, we examined the impacts of a specific inhibitor of ferroptosis, ferrostatin-1 (Fer-1) on WS2 and MoS2 induced cell deaths. As shown in Figure 4A, Fer-1 pretreatment could dramatically improve cell viabilities (24-47% increments) in BEAS-2B and THP-1 cells exposed to WS2 and MoS2.”

**WS2	MoS2	WS_2_	MoS2**

120

Fer-1 (-) Fer-1 (+)

*

***

100

80

60

40

**Cell viability by MTS (%)**

*

**

20

0

**BEAS-2B cell	THP-1 cell**

**Figure R1 Effects of Fer-1 on MoS2 and WS2 induced cytotoxicity**

BEAS-2B or THP-1 cells were pretreated by 10 μM Fer-1 for 1 h, followed by 48 h incubation with 200 μg/mL WS2/MoS2 (n=3). MTS assay was used to assess the impacts of Fer-1 on WS2/MoS2-induced cell deaths. * p< 0.05, ** p< 0.01, *** p< 0.001 compared to cells without Fer-1 treatment by two tailored Student T-test.

1

2. Bubbling is one of the common phenotype on lipid peroxidation mediated ferroptotic cell death. It would be good to check the bubbling phenotypes in BEAS-2B cells when cell death caused by WS2 and MoS2 nanoparticles.

**Response:** Thanks for the suggestion. We examined the morphology changes of BEAS-2B cells exposed to WS2 and MoS2 for 48 h. As shown in Figure R2, TMD nanosheet treatments induced cell swelling, which is different to cell shrinking in apoptosis induced by DOX. These images are consistent with the cell growth movie (Movie S1 and S3) as well as literature reports (J Cell Mol Med. 2017, 648-657).

**Figure R2 Morphologies of TMD-treated BEAS-2B cells**

Ctrl	DOX	WS2	MoS2

BEAS-2B cells were exposed to 2 μg/mL DOX, 200 μg/mL WS2 and MoS2 nanosheets for 48 h. The cell morphologies were examined by a light microscope (Scale bar represents 50 μm).

3. To claim WS2 and MoS2 nanoparticles induced ferroptotic cell death, authors further validate some ferrototic markers like PTGS2, GPX4 etc protein and mRNA expression.

**Response:** Thanks for the reviewer’s suggestions. We examined the expression of GPX4 protein in BEAS-2B cells by Western blotting. As shown in Figure R3, WS2 and MoS2 treatments significantly suppressed the abundance of GPX4 (Figure R3). This result has been added in revised manuscript as Figure 4B, and described in line 200-204, as “A reduction of glutathione peroxidase 4 (GPX4) in response to WS2 and MoS2 nanosheets was confirmed by Western blotting (Figure 4B). Immunoblotting to examine the abundance of GPX4 in BEAS-2B extracts demonstrated that WS2 and MoS2 treatments suppressed >30% expression of GPX4 in cells.”

2

1.2

|  |  | **BEAS-2B cells** |  | **Relative expression of GPX4** | 1 |
| --- | --- | --- | --- | --- | --- |
|  | Ctrl WS2 MoS2 | | |  |  |
| **GPX4**  **β-Actin** |  | | |  | 0.8  0.6  0.4  0.2 |

*

*

Ctrl	WS2	MoS2

0

**Figure R3 Assessment of GPX4 expression in BEAS-2B cells.**

BEAS-2B cells were exposed to 200 μg/mL WS2/MoS2 for 48 h, and then the cells were collected, washed and lyzed for Western blotting assay (left panel). The relative expression of GPX4 was assessed by the optical density of each band on Western blotting images (right panel, n=3). *p <0.05 compared to ctrl cells by two tailored T-test.

4. Figure 6, authors measured most of the inflammatory markers in tissue. It would be ideal to check ferroptosis markers in isolated lung tissue.

**Response:** Thanks for the suggestion. To accommodate the reviewer’s concern, we examined more biomarkers of ferroptosis in animal lungs, including Fe^2+^ release, lipid peroxidation and GPX4 expression. As shown in Figure R4, reduction of GPX4, release of Fe^2+^ and lipid peroxidation were detected in alveolar macrophages extracted from WS2-treated mouse lungs, but the abundance of GPX4 in whole lung tissue had limited changes. Notably, Fer-1 treatment could ameliorate these effects (Figure R4). These results have been added in revised manuscript as Figure 6, and described in line 266-275, as “First of all, we examined GPX4 expression, Fe^2+^ release and lipid peroxidation to validate the ferroptosis mechanism. As shown in Figure 6A, reduction of GPX4 was identified in alveolar macrophages extracted from animal lungs exposed to WS2 nanosheets. Meanwhile, WS2 treatments led to significant Fe^2+^ leakage in alveolar macrophages by FeRhoNox-1 staining, displaying diffused red fluorescence in whole cytoplasm (Figure 6B). Subsequently, the released Fe^2+^ could elicit lipid peroxidation, evidenced by a majority of alveolar macrophages displaying strong green fluorescence of oxidized BODIPY C11. Notably, Fer-1 treatment could ameliorate the impacts of WS2 on GPX4 expression, and suppressed Fe^2+^ release and lipid peroxidation in alveolar macrophages.”

3

**A	B**

**Confocal imaging of Fe^2+^ in alveolar macrophages**

Ctrl Fer+WS_2_ WS_2_

| **GPX-4**  **β-actin** |  |
| --- | --- |

**C**

| Ctrl | WS2 | Fer-1 + WS2 |
| --- | --- | --- |
| Blue: Nucleus  Red: Fe^2+^ |  |  |

**Lipid peroxidation imaging in alveolar macrophage**

**Nuclei	BODIPY C11 Oxidized BODIPY C11	Overlay**

**Figure R4 Validation of ferroptosis in alveolar macrophages from lung tissue**

Ctrl

WS2

Fer+WS2

**A)** Western blotting images of GPX-4, confocal imaging of **B)** Fe^2+^ release and **C)** lipid peroxidation in alveolar macrophages. Animals pretreated with or without 50 jig/Kg Fer-1 for 2 h were exposed to 1 mg/Kg WS2 for 40 h (n=6). Then the BALF of animals were collected to extract alveolar macrophages. The cells were divided into three portions. One was lysed for Western blotting analysis, and the other two were stained by Image-iT lipid peroxidation staining kit and FeRhoNox-1for confocal imaging (scale bar: 10 jim).

5. There are numerous grammatical and typographical errors throughout the paper. Please go through overall the manuscript.

**Response:** Thanks for the suggestion. We have double checked the whole manuscript and corrected these errors in line 22, 39, 61, 63, 76, 78, 81, 87, 91, 94, 97-100,

| 109-110, | 116-118, 128, 131, 139-140, 142, 145, 144, 160, 165-166, 170-171, | 173, | 175, |
| --- | --- | --- | --- |
| 179, 186, | 190, 207, 211-212, 254, 281, 285-286, 291, 293, 298, 307, 325, | 371, | 375, |
| 395, 734, | 735-740. |  |  |

**Reviewer: 2**

Comments:

1. The manuscript of S. Xu at all show the influence of MoS2 and WS2 on cell

4

metabolism. The interaction of layered disulfide was reported by several authors in recent years. I don’t see sufficient significance for publication of this work in Nature Communication.

**Response:** Thanks. We agree with the reviewer that there are several studies on the interactions between TMDs and biological systems. A few representative studies have been discussed in our manuscript (Nat. Nanotechnol. 12, 343-350; Nanoscale 6, 14412-14418; Small 11, 5079-5087). However, compared to literature reports, our study made two innovative findings: i) MoS2 and WS2 were able to induce ferroptosis in cells and animal lungs; ii) the vacancy on nanosheet surfaces was responsible for the ferroptotic cell deaths. To the best of our knowledge, we defined the first report of 2D TMD induced ferroptosis as well as the identification of key physicochemical properties responsible for this effect. We added a sentence (line 303-306) in discussion section to emphasize the novelty of this study, as “Compared to other nano-bio studies on 2D TMDs, our study made two interesting findings: i) MoS2 and WS2 were able to induce ferroptosis in cells and animal lungs; ii) the vacancy on nanosheet surfaces was responsible for the ferroptotic cell deaths.”

2. In the manuscript are also not sufficient characterization of exfoliated MoS2/WS2. There is none clear evidence of vacation formation. This should be shown by HR-TEM in combination with other methods like XPS and other techniques which provide sufficient evidence of vacancies in chalcogenides sheets. I recommend to publish this work in more specialized journal.

**Response:** We thank the reviewer for these suggestions. We used HR-TEM to visualize the vacancies on MoS2 and WS2 nanosheets. As shown in Figure R5, while substantial single vacancies and linear surface vacancies could be identified on the surfaces of WS2 and MoS2 nanosheets (yellow circles), surface passivation is an effective strategy for vacancy healing. This result is consistent with a report by Zhang et al. (Nature Communication, 2017, 8, 15881). The HR-TEM images of vacancies are added in revised manuscript as Figure S7, and described in line 234-238, as “We exploited high resolution (HR) TEM to visualize the vacancies on pristine and functionalized MoS2 and WS2 nanosheets. As shown in Figure S7, while substantial single vacancies and linear surface vacancies could be identified on the surfaces of WS2 and MoS2 nanosheets (yellow circles), surface passivation is an effective strategy for vacancy healing.”

5

HR-TEM

**Figure R5 HR-TEM visualization of vacancies on pristine and modified TMD surfaces**

WS2	MoS2

ss-WS2	me-MoS2	WSe2

WS2, MoS2, ss-WS2, me-MoS2 and MoSe2 nanosheets were dropped on ultra-thin carbon film to visualize atom arrangement by HR-TEM (Titan Cubed, Thermo Fisher Scientific) under an electron beam flux of ~50 A/cm^2^ at 80 kV. Irregular yellow circles indicate surface vacancies. The scalebar is 1 nm.

**Reviewer: 3**

Comments:

Xu et al. have studied a panel of five different 2D materials with respect to cytotoxicity and show that 2 of the materials namely WS2 and MoS2 elicit ferroptosis, an iron-dependent form of cell death in the human THP-1 and BEAS-2B cell lines in vitro). The authors have also documented a pro-inflammatory response to these materials in an animal model following pharyngeal aspiration, but did not confirm ferroptosis in vivo.

**Response:** We thank the reviewer for the thorough evaluation of our work, and raised suggestions to improve our study Based on the reviewer’s suggestions, we have performed more experiments to validate the ferroptosis effects in animals. We hope the new results could improve our study significantly.

1 The authors must carefully attend to the following major remarks:

1.1 The authors have overstated the conclusion regarding the role of metal vacancies on WS2 and MoS2 surfaces (cf. the title of the manuscript). The study shows that passiviation of these materials by saturation with Na2S and methanol, respectively, results in a partal (yet significant) reduction in cytotoxicity. However, more detailed studies are required to prove that metal vacancies are playing a key role.

**Response:** To address the reviewer’s concern on the role of vacancies in ferroptosis, we first used HR-TEM to directly visualize vacancies on the surfaces of TMD nanosheets. As shown in Figure R5 (please fine it in our response to reviewer 2), we visualized substantial single vacancies and linear surface vacancies on the surfaces of WS2 and MoS2 nanosheets (yellow circles), whereas ss-WS2, me-MoS2 and MoSe2 showed very limited vacancies. These images indicated that surface passivation is an

6

effective strategy for vacancy healing. Furthermore, we prepared 20 derivatives of TMD nanoparticles by separating suspensions of TMD exfoliates at different centrifugations (Gao *et al*, Angew Chem, 2020, 59, 3618). We characterized the physicochemical properties of these TMD nanoparticles. The surface atom ratios of S/Se *v.s.* Mo/W determined by XPS were able to reflect the vacancy densities (Chee *et al*, Nanoscale, 2017, 9333; Sim *et al*, ACS Nano, 2015, 12115). As shown in Table R1, these nanoparticles had different compositions, sizes, surface charges and surface atom ratios. We examined the correlations between cell viability and physicochemical properties of TMDs. Pearson correlation analysis showed that surface atom ratio had strong correlation with cell viability, displaying a correlation factor of |r| at 0.89. We believe these data well support our statement regarding the role of vacancies in WS2 and MoS2 induced cytotoxicity. This table was added in revised manuscript as Table 1, and described in line 214 to 227, as “Since the active Mo or W atoms resulting from surface vacancies were found to take part in the MIEs of ferroptosis, we hypothesized that the surface vacancy may be the key physicochemical property responsible for the ferroptosis effect of MoS2 and WS2 nanosheets. To demonstrate this hypothesis, we prepared 20 TMD nanoparticles to examine the impacts of vacancy on cell viability as nano-SARs are often determined by *in silico* analysis of quantitative descriptors of intrinsic properties and biological receptors acquired from sufficiently large databases of nanomaterials 27, 28. The vacancy densities of TMDs were assessed by surface atom ratios of S/Se *v.s.* W/Mo29, ^30^. As shown in Table 1, these nanoparticles had different compositions, sizes, surface charges and surface atom ratios. We examined the correlations between cell viabilities and physicochemical properties of TMDs. Pearson correlation analysis showed that surface atom ratio (vacancy density) had strong correlation with cell viability, displaying a correlation factor of |r| at 0.89. This result indicated that surface vacancy may be the critical property dictating the ferroptosis effect induced by WS2 and MoS2 nanosheets.”

7

**Table R1 Correlation analysis between cell viability and physicochemical property**

| **TMD** | **Physicochemical properties** | | | | | **Cell  viability**  **(%)** |
| --- | --- | --- | --- | --- | --- | --- |
|  | Thickness  (nm) | Diameter (nm) | Hydrodynamic size (nm) | Zeta  potential  (nm) | Surface  atom ratio* |  |
| 1 | 211±8 | 384±9 | 189.7±0.4 | -20.7±0.4 | 1.77 | 108.4 |
| 2 | 90±3 | 178±5 | 139.5±3.2 | -24.5±1.5 | 1.82 | 117.4 |
| **WS2** 3 | 40±2 | 108±3 | 120.2±0.4 | -24.7±1.3 | 1.75 | 108.7 |
| 4 | 2±1 | 10±2 | 94±2 | -6±0.3 | 1.21 | 54.6 |
| 5 | 2±1 | 12±1 | 96±1 | -7.2±2 | 1.77 | 89.2 |
| 1 | 153±6 | 407±2 | 308.7±1.8 | -25.4±1.2 | 1.71 | 113.9 |
| 2 | 79±3 | 199±5 | 143.9±0.2 | -24.8±0.8 | 1.70 | 114.2 |
| **MoS2** 3 | 49±3 | 175±1 | 115.3±0.9 | -27.5±0.4 | 1.53 | 91.5 |
| 4 | 3±1 | 9±3 | 91±1 | -18.6±0.3 | 1.31 | 57.3 |
| 5 | 3±1 | 10±2 | 93±2 | -17.5±1.0 | 1.65 | 101.2 |
| 1 | 202±8 | 304±6 | 204±4 | -20.7±0.4 | 1.65 | 105.3 |
| 2 | 140±4 | 92±2 | 146±4 | -27.8±0.7 | 1.71 | 116.9 |
| **WSe2** 3 | 42±8 | 78±4 | 150±5 | -20.3±0.2 | 1.60 | 99.9 |
| 4 | 16±4 | 62±6 | 106±0.5 | -23.5±0.1 | 1.61 | 96.2 |
| 5 | 2±1 | 14±3 | 94±1 | -24.2±0.3 | 1.64 | 99.4 |
| 1 | 160±2 | 232±2 | 349±9 | -21.5±0.3 | 1.73 | 100.5 |
| 2 | 91±7 | 113±9 | 152±1 | -20.9±0.1 | 1.80 | 112.9 |
| **MoSe2** 3 | 39±7 | 73±9 | 145±3 | -20.4±0.2 | 1.81 | 115.3 |
| 4 | 21±4 | 50±1 | 120±5 | -20.2±0.2 | 1.71 | 98.4 |
| 5 | 2±1 | 16±4 | 123±2 | -18.7±0.4 | 1.68 | 92.8 |
| **Correlation** |  |  |  |  |  |  |
| **factor** | 0.51 | 0.48 | 0.38 | -0.64 | 0.89 | NA |

* represents the ratio of S/Se *v.s.* W/Mo determined by X-ray photoelectron spectroscopy (XPS).

1.2 Furthermore, the suggestion that this amounts to "safe design" is questionable: a material can elicit more or less cytotoxicity but "safe-by-design" implies that a material retains its useful properties while displaying a reduced toxicity; if a material is inert and no longer displays useful properties then this is passivation but not "safe design".

**Response:** We agree with the reviewer that “safe design” implies a material retains its useful properties while displaying a reduced toxicity. Since TMDs were extensively used as lubricant, we performed a ware test on WS2, ss-WS2, MoS2 and me-MoS2. As shown in Figure R6, surface modified WS2/MoS2 showed similar friction coefficients to pristine materials. Therefore, from the perspective of lubricant uses, the surface

8

passivation strategies developed in this study could be considered as a safe design approach. This result has been added in revised manuscript as Figure S8, and described in line 237 to 242, as “Before biocompatibility tests, we first examined whether the surface modifications may affect the lubricating properties of TMDs as they are often used as lubricant additives. As shown in Figure S8, Na2S and methanol treatments had negligible effects on the friction coefficients of WS2 and MoS2, respectively. We therefore thought the surface passivation didn’t affect the utility of TMDs as lubricants.”

|  | 0.1  0.08  0.06 |
| --- | --- |
| **Friction coefficient** |  |
|  |  |
|  | 0.04  0.02  0 |
|  |  |

**Figure R6 Friction coefficients of pristine and functionalized MoS2 and WS2** Lubricant (100 mg) was added to the contact area on an Optimal-SRV-IV tribometer (SRV load, 100 N; temperature, 25 °C; stroke, 1 mm; frequency, 25 Hz). The friction coefficient was recorded automatically *via* a computer connected to the SRV tester.

1.3 Does the passivation also work in vivo?

**Response:** Yes. First, we examined the impacts of surface passivation on ferroptosis in animal lungs. As shown in Figure R7, Na2S treatment remarkably reduced the Fe^2+^ release and lipid peroxidation in alveolar macrophages extracted from mouse lungs. In addition, we examined the neutrophil counts and cytokine release in BALF as well as inflammation in mouse lungs receiving oropharyngeal instillation of WS2, MoS2, ss-WS2 and me-MoS2. As shown in Figure 7, compared to pristine WS2 and MoS2, surface modified nanosheets (ss-WS2 and me-MoS2) had negligible effects on neutrophil counts and cytokine production in BALF, and induced limited focal areas of inflammation in lung tissue. All these results demonstrated that the surface passivation approaches also worked *in vivo*. Figure R7 has been added in revised manuscript as Figure S12.

9

**A	B**

**Lipid peroxidation staining in alveolar macrophage**

**Nuclei	BODIPY C11 Oxidized BODIPY C11	Overlay**

**Figure R7 Impacts of surface passivation on WS2 induced ferroptosis in alveolar macrophages**

**Confocal imaging of Fe^2+^**

ss-WS2

Ctrl

Blue: Nucleus Red: Fe^2+^

PBS

ss-WS2

Confocal imaging of **A)** Fe^2+^ and **B)** lipid peroxidation in alveolar macrophages extracted from mouse lungs. Animals received oropharyngeal aspiration of 1 mg/Kg ss-WS2 were sacrificed at 40 h to collect macrophages from BALF (n=3). The alveolar macrophages were seeded in eight-well chamber and stained by FeRhoNox-1 and Image-iT lipid peroxidation staining kit to visualize Fe^2+^ and lipid peroxides by confocal microscopy, respectively (scale bar: 10 μm).

1.4 How stable or long-lasting is this surface saturation with methanol or Na2S? **Response:** Thanks for raising this critical question. We evaluated the stability of Na2S and methanol modifications by comparing the viabilities of BEAS-2B cells exposed to fresh-made and aged ss-WS2 and me-MoS2 in DI water. As shown in Figure R8, the fresh-made and five-month aged ss-WS2 and me-MoS2 exhibited similar cell viabilities. We therefore concluded that surface modification is able to stably exist more than five months.

**Figure R7 Stability assessment of surface saturation**

100

80

60

40

20

**BEAS-2B cell viability by MTS (%)**

0

WS2 MoS2

BEAS-2B cells were exposed to 200 μg/mL ss-WS2 and me-MoS2 stored for 1 day, 7

day, 1 month, 2 month, 4 month and 5 month. The cell viability was examined by

10

MTS assay.

1.5 Does the passivation selectively affect ferroptosis or is it possible that other modes of programmed or non-programmed cell death also contribute to the cytotoxcity effects of the TMDs?

**Response:** Thanks for raising this question. We think the passivation developed in this study may be a general protection strategy to ameliorate cytotoxicity of TMDs, rather than a selective reduction of ferroptosis. Although ferroptosis is the dominating mechanism for cell deaths induced by WS2 and MoS2 nanosheets in our study, it’s likely that other programmed or non-programmed cell death may also contribute to the cytotoxicity of TMDs. This is because we cannot rule out following possibilities: i) WS2 and MoS2 may induce other programmed and non-programmed cell deaths in other cell lines; ii) other untested TMDs may induce different cell deaths.

2. Abstract

2.1 The abstract is deficient in that the authors failed to mention that they have studies five different TMDs.

**Response:** Thanks for the suggestion. We have modified the abstract and added the number of TMDs, as “Herein, we explored the toxic effects of five different 2D transition metal dichalcogenides (TMDs)...”.

2.2 they do not mention that the work was carried out using the BEAS-2B and THP-1 cell model.

**Response:** Thanks for the suggestion. We have described the cell models in Abstract, as “WS2 and MoS2 nanosheets induced an iron-dependent cell death pathway, ferroptosis in epithelial (BEAS-2B) and macrophage (THP-1) cell lines”.

2.3 The authors also need to carefully reassess the use of the term "nano-SARs" (or structure-activity relationship studies). One cannot extract information on structure-activity relationships of a class of materials based on the study of only 2 materials. Achieving the description of nano-SARs or informative nano-descriptors with which to predict toxicity of nanomaterials is a formidable task and one that requires studies of sufficiently large databases of nanomaterials of reasonable diversity with respect to intrinsic properties and biological receptors; most often, computational approaches are required (Puzyn T et al. Small. 2009;5(22):2494–2509; Liu R et al. 2011;7(8):1118-1126). The present study is NOT a nano-SAR study.

**Response:** Thanks for the reviewer’s comments. Based on the recommended nano-SAR analysis methods (Puzyn T et al. Small. 2009, 2494; Liu R et al.

11

2011,1118), we increased the database of TMD materials for nano-SAR assessment in this study. We synthesized 20 TMD nanomaterials with different heights, surface charge, sizes, and surface atom ratios by a reported method (Gao *et al*, Angew Chem, 2020, 3618). We quantitatively characterized the physicochemical properties of these nanosheets. An *in silicon* Pearson correlation analysis by SPSS software was used to identify the key physicochemical properties responsible for TMD-induced cell deaths (Cai *et al*, Nature Communications, 2018, 4416). As shown in Table R1, the surface atomic ratios reflecting vacancy densities showed the best correlation with ferroptotic cell deaths of all 20 materials. We hope these results significantly strengthen our claim on nano-SAR in the revised manuscript.

3. Introduction

3.1 This section is very poorly written and hardly provides sufficient background information for the present study. The very first sentence is very ambiguous and seems to suggest a curious circular argument instead of simply stating that it is important to understand which properties that are driving the toxicity of nanomaterials;

**Response:** Thanks for your comments. We have made some changes in the first sentence, as “As physicochemical properties of engineered nanomaterials (ENMs) were found to significantly impact their toxicities...”

3.2 The first paragraph is focused on SARs but this is not relevant for the current study. The authors also failed to provide a relevant background on previous studies of 2D materials thus giving the impression that the current study is the first to address the potential toxicity of this class of materials but this is certainly no correct.

**Response:** Thanks for the comments. We have made three major changes in revised manuscript to accommodate the reviewer’s concern. First, as responded in 2.3, we have added new data to strengthen our claim on nano-SAR of 2D TMDs. We think a brief introduction of SARs is relevant to our study. Second, we have made some changes in revised manuscript to claim that our study defined the first report on TMD-induced ferroptosis effect in mammals (The first sentence in conclusion), rather than “the current study is the first to address the potential toxicity of this class of materials”. Finally, we have added several representative studies on TMD toxicity in the first paragraph of introduction, as “Recently, two-dimensional (2D) transition metal dichalcogenides (TMDs) as an atomic-layer semiconductor are found to display unique surface activities^7^ and arouse massive research enthusiasms on their biological effects. The surface area, active edge sites and aggregation states were found to impact the hazard effects of TMDs in cells and animals^8-11^. For instance, Wang et al

12

discovered that aggregated MoS2 induced proinflammatory cytokine release in cells and acute lung inflammation in mice, whereas dispersed MoS2 had little effects^9^.” we hope these revisions have significantly improved the quality of introduction section.

3.3 Some relevant references are reported in the discussion (page 13) but this short paragraph also fails to provide all the relevant information, and there are certainly many other reasons for the "conflict toxicity results" including not only "different sources" of the TMDs but also the use of different in vitro or in vivo models, different doses, and so on.

**Response:** Thanks for your suggestions. We have revised this section to include more relevant studies and discussed the impacts of biological model, ENM doses, incubation time, exposure routes on the conflict toxicity of TMDs. The discussions have been added in line 319 to 332, as “The differences of material source, physicochemical properties of TMDs, exposure time, doses and routes may be responsible for the conflict reports. Wang *et al* used same cell lines (THP-1 and BEAS-2B) to us for cytotoxicity assessments and found limited cell viability changes at 24 h incubation with 0-50 μg/mL MoS2 nanosheets, whereas we merely observed significant cytotoxicity at 48 h incubation with 50-200 μg/mL MoS2 nanosheets, indicating that the exposure time and doses of TMDs may greatly affect their cytotoxicity. In terms of the impacts of exposure routes, Mei *et al*’ study showed that intravenous injected MoS2 elicited more toxicity than intraperitoneal and intragastric administration^11^. Under similar doses, same exposure time and route, we and Wang *et al* discovered similar pulmonary inflammation effects for MoS2 nanosheets. Besides, consistent with our nano-SAR findings, the surface chemistry of TMDs may play a major role in their toxicities, evidenced by the increased biocompatibility of MoS2 nanosheets functionalized by Pluronic 127^42^, Pluronic 87^9^ and PEG molecules^43^.”

3.4 The authors also exaggerate the approach taken in the present study: one cannot refer to a small panel of 5 materials as a "library" of TMDs. Studies using 81 different surface-modified MWCNts (Wu L et al. ACS Nano. 2014 Mar 25;8(3):2087-99) or 105 surface-modified gold NPs (Walkey CD et al. ACS Nano. 2014 Mar 25;8(3):2439-55) are considered as studies of "libraries" but the present study is on a small panel (n=5) of TMDs

**Response:** Thanks for the reviewer’s comments. We have revised the manuscript to avoid the use of “library” for description of a small panel of materials.

3.5 it is NOT a nanoSAR study;

**Response:** Thanks for the reviewer’s comments. As we responded in 1.1 and 2.3, we

13

have prepared a large number of TDM nanomaterials and explored the structure-activity relationships by *in silicon* methods. We hope our new data significantly strengthen our claim on the nano-SAR of 2D TMDs.

3.6 no predictive information can be derived of the study of 2 different TMDs (with or without surface passivation).

**Response:** Thanks for the reviewer’s comment. We have expanded the number of TMD materials for nano-SAR analysis. Based on this information, we speculated that the hazard ranking of TMD materials could be predicted by surface vacancy densities.

3.7 Again, the "safer design" of these materials is also questionable as the authors did not test the materials for their functionality, only whether or not they caused cytotoxicity.

**Response:** Thanks. As responded in comment 1.2, we tested the impacts of surface passivation of WS2 and MoS2 on their use as lubricants. As a result, Na2S and methanol treatments had negligible effects on friction coefficients, and did not affect the lubricating properties of WS2 and MoS2. We therefore think the surface passivation methods in this study could be claimed as a safe design approach from the perspective of TMD application as lubricants.

4. Results

4.1 The authors have used very high doses of the TMDs (up to 200 ug/mL). What is the relevance of using such high doses?

**Response:** Thanks for pointing out this critical question. Considering the OSHA permissible exposure thresholds, soluble and insoluble molybdenum materials in workroom air may reach to 5 and 15 mg/m^3^, respectively. Based on a calculation approach being used at NIOSH (Particle and Fibre Toxicology, 2017, 14:13), we could estimate that a worker exposure to 15 mg/m^3^ TMDs for 8 h/day over a five-month time period over a 5-year working time could lead to a lung burden of 1.875 mg/m^2^. Assuming a homogeneous distribution of TMD in tissue culture dish and 10 μm thickness of cell layer (ACS Nano, 2013, 7, 3, 2352-2368), the corresponding *in vitro* TMD dose would be 0-187.5 μg/mL. Therefore, the *in vitro* TMD doses used in this study are setting at 0-200 μg/mL. We have justified the *in vitro* and *in vivo* doses in line 339 to 352, as “Since TMDs are popularly used as lubricants in industries and daily life, the fine particulates of TMDs in aerosols have high risks of inhalation exposure. The OSHA permissible exposure thresholds for soluble and insoluble molybdenum materials in workroom air are set at 5 and 15 mg/m^3^, respectively^44^. Based on a calculation approach being used at NIOSH^45^, we

14

could estimate that a worker exposure to 15 mg/m^3^ TMDs for 8 h/day over a five-month time period over a 5-year working time could lead to a lung burden of 1.875 mg/m^2^. Considering that a bolus exposure of 1 mg/kg in the mouse is equivalent to 0.5 mg/m^2^ of nanoparticle exposure in mouse lungs, oropharyngeal aspiration of 0-3.6 mg/Kg TMDs in mice is comparable to the real exposure levels of TMDs in workroom air. Assuming a homogeneous distribution of TMD in tissue culture dish and 10 μm thickness of cell layer^44^, the corresponding *in vitro* TMD dose would be 0-187.5 μg/mL. Overall, the *in vitro* (0-200 μg/mL) and *in vivo* (1 mg/Kg) doses used in this study is relevant to the possible occupational exposure in real scenarios.”

4.2 For the in vivo studies, the authors applied 1 mg/mg of TMDs. What is the relation between the in vitro dose and the in vivo dose in the present study?

**Response:** Thanks for the reviewer’s comments. We have justified the relationship of *in vitro* dose and animal dose in line 339 to 352. Please refer our response to comment 4.1.

4.3 how does the in vivo dose compare with the doses that may potentially occur in a human setting?

**Response:** Thanks for raising this question. We have discussed the relevance of *in vivo* dose with potential exposure levels in humans in revised manuscript (line 339 to 352). Please refer our response to comment 4.1.

4.4 Minor comment: the authors write that the 2D materials were exposed to the THP-1 and BEAS-2B cells, but it is in fact the other way around.

**Response:** Thanks for pointing out this error. We have corrected this in the revised manuscript.

4.5 Overall, the author may want to check the manuscript for grammatical consistency (for instance, the first sentence of the main text: "nanobiology researches", and so on). **Response:** Thanks. We have double-checked the grammar errors in whole manuscript and made corrections in line 22, 39, 61, 63, 76, 78, 81, 87, 91, 94, 97-100, 109-110,

| 116-118, 128, | 131, 139-140, | 142, | 145, 144, | 160, | 165-166, | 170-171, | 173, | 175, | 179, |
| --- | --- | --- | --- | --- | --- | --- | --- | --- | --- |
| 186, 190, 207, | 211-212, 254, | 281, | 285-286, | 291, | 293, 298, | 307, 325, | 371, | 375, | 395, |
| 734, 735-740. |  |  |  |  |  |  |  |  |  |

4.6 The suggestion that all materials have "similar dosimetry effects in their interactions with cells" is not supported by the data. What the authors have shown is that the materials are well dispersed in cell medium, but the density of each material

15

will determine the in vitro dosimetry, and there are no "dosimetry effects".

**Response:** Thanks for pointing this out. We have deleted the description on dosimetry

effects in the revised manuscript.

4.7 To fully understand the intracellular dose, the authors need to check cellular uptake of each material.

**Response:** Thanks for the reviewer’s comment. We detected the cellular uptake of each material by ICP-MS. As shown in Figure R8, all five materials had similar cellular internalization (30-40 μg/mg protein) in BEAS-2B cells.

**Metal ion content (μg/mg protein)**

45

40

25

20

50

35

30

15

10

5

0

**Figure R8 Cellular uptake of 2D TMDs in cells**

BEAS-2B cells in six-well plates were exposed to 2D TMDs at the concentration of 100 μg/mL for 24 h, followed by cell washing, lysis and digestion (n=3). The intracellular uptake of TMDs were determined by ICP-MS detection of W, Mo and B elements.

4.8 Concerning the mode of cell death: The authors have shown that zVAD-fmk (a pan-caspase inhibitor, not an "apoptosis inhibitor") and necrostatin-1 failed to block cell death. Why did the authors not test the commonly used ferroptosis inhibitor, ferrostatin-1? Why did test only NAC and not, for instance, Trolox? The results suggest a role for ROS, but more evidence is needed to prove that this is ferroptosis. **Response:** Thanks for these great suggestions. Some of these suggestions are coincidently similar to the concerns by reviewer 1. We have performed new experiments based on these suggestions.

First, we examined the effects of ferrostatin-1 (Fer-1) in cells and animal lungs. These new results have been added in revised manuscript as Figure 4A and 6. As shown in Figure 4A, Fer-1 could significantly ameliorated the cell deaths induced by WS2 and

16

MoS2; In animal lungs, Fer-1 treatments could ameliorate the reduction of GPX4 (a biomarker of ferroptosis), and significantly prevent Fe^2+^ release as well as lipid peroxidation in alveolar macrophages (Figure 6).

Second, a biomarker of ferroptosis (GPX-4) was examined in BEAS-2B cells and animal lungs. As shown in Figure 4B and 6A, WS2 suppressed the expression of GPX-4 in BEAS-2B cells and alveolar macrophages.

Third, the effect of Trolox was tested in TMD-treated cells. As shown in Figure R9, Trolox could significantly ameliorated the cell deaths of BEAS-2B cells exposed to WS2 and MoS2. Figure R9 has been integrated in Figure S5 of revised manuscript. We hope these new results significantly strengthen our claim on ferroptosis.

**WS2	MoS2**

140

**BEAS-2B cell viability by MTS (%)**

120

100

40

20

80

60

0

Trolox (-)

Trolox (+)

*

**

**Figure R9 Effects of Trolox on WS2/MoS2 induced cell deaths**

BEAS-2B cells pretreated with 50 μM Trolox for 1 h were exposed to 200 μg/mL WS2 and MoS2 for 48 h to determine cell viability by MTS assay. *p<0.05, ** p< 0.01 compared to cells without Trolox pretreatment.

4.9 Furthermore, why did the authors not check for cell death/ferroptosis in the animal model? The authors claim that the results obtained in the animal model validated or confirmed the in vitro results but this is not correct. The in vitro studies were focused on cell death, lipid peroxidation, lysosomal involvement while the in vivo study addressed pro-inflammatory effects in the lungs of exposed mice.

**Response:** Thanks for the reviewer’s comments. This comment is also coincidently similar to the comment 4 by reviewer 1. To further examine the ferroptosis in animals, we detected GPX4 expression, Fe^2+^ release as well as lipid peroxidation in mouse lungs exposed to WS2. WS2 suppressed the expression of GPX4 in alveolar macrophages (Figure 6A). In addition, WS2 was able to elicit Fe^2+^ release and lipid

17

peroxidation in alveolar macrophages from BALF (Figure 6B and 6C). Fer-1 treatment could ameliorate the reduction of GPX4, and attenuate Fe^2+^ release and lipid peroxidation in alveolar macrophages. These results well validated TMD-induced ferroptosis in animal lungs. A description of these results has been added in line 265-274, as “First of all, we examined GPX4 expression, Fe^2+^ release and lipid peroxidation to validate the ferroptosis mechanism. As shown in Figure 6A, reduction of GPX4 was identified in alveolar macrophages extracted from animal lungs exposed to WS2 nanosheets. Meanwhile, WS2 treatments led to significant Fe^2+^ leakage in alveolar macrophages by FeRhoNox-1 staining, displaying diffused red fluorescence in whole cytoplasm (Figure 6B). Subsequently, the released Fe^2+^ ions could elicit lipid peroxidation, evidenced by a majority of alveolar macrophages displaying strong green fluorescence of oxidized BODIPY C11. Notably, Fer-1 treatment could ameliorate the impacts of WS2 on GPX4 expression, and suppressed Fe^2+^ release and lipid peroxidation in alveolar macrophages.”

4.10 The authors used cytochalasin D to show that the toxicity of the TMDs was significantly reduced and they draw the conclusion that this "suggests that lysosome internalized WS2 and MoS2 are responsible for the downstream hazard signals" but this is also incorrect. This experiment shows that endocytosis is required for cytotoxicity but does not give any clues regarding the lysosomes.

**Response:** Thanks for the reviewer’s comments. We have made changes on this conclusion as “These data suggested that endocytosis was required in cell deaths induced by WS2 and MoS2”.

4.11 The results obtained with NAC (a precursor of GSH) are important. But can the authors exclude that NAC is acting as a metal chelator and not (only) as an antioxidant?

**Response:** To the best of our knowledge, there are currently deficient techniques to clearly differentiate the role of NAC acting as a metal chelators or antioxidant in a complex cellular environment. However, we think NAC works more like an antioxidant rather than a metal chelate based on following results. First of all, we examined the dissolution of TMDs in cell culture media and phagolysosome simulated fluid (Li et al, ACS Nano, 2014, 1996). WS2 and MoS2 showed only 3% and 1% dissolution, respectively. This data indicated that metal ions released from WS2 and MoS2 were very low in biological contexts. Second, we have demonstrated that the released W and Mo ions had limited effects on cell viability (Figure S3). Third, we found Trolox exhibited similar protection effects to NAC in TMD-treated cells (Figure S5).

18

4.12 The results obtained with the 2 iron chelators are also important. Again, can the authors conclusively show that these reagents bind iron (a key finding on which the entire ferroptosis story is based) as opposed to chelating other metals including the TMDs themselves?

**Response:** Thanks for raising this question. As we mentioned above, there are currently deficient techniques to directly examine the metal ions interacting with iron chelators in complex cellular compartments. However, we think the detoxification effect of iron chelators is due to their interaction with iron ions rather than the metal ions released from TMDs. This is because: i) the levels of W/Mo ions in biological contexts are very low and not responsible for ferroptosis; ii) the binding constants of chelators (e.g. DFX) with irons (LogK ~ 37) are significantly higher than tetravalent metal ions (LogK ~ 26) (Inorg. Chem. 1996, 4128-4136; Blood, 2017, 1923-1933).

4.13 Overall, the authors speculate too much in the results section. For instance, they claim that the experiments using PC liposomes and red blood cells (hemolysis assay) "indicate" that metal vacancies on MoS2 and WS2 "may affect the architectures of lysosomes and elicit lysosomal membrane permeabilization (LMP) by oxidative damages or phospholipid extraction". But this was not proven by the latter experiments, this is an extrapolation.

**Response:** Thanks the reviewer for pointing this out. To address the reviewer’s concern, we added new data in the revised manuscript. First, our new results clearly showed that there are substantial vacancies on pristine MoS2 and WS2 surfaces by HR-TEM (Figure S7), whereas surface modified nanosheets (ss-WS2 and me-MoS2) had limited vacancies. Second, the nano-SAR analysis also indicated that vacancy densities had strong correlation with ferroptotic cell deaths (Table 1). Third, we used a lysosomal isolation kit to acquire lysosomes from BEAS-2B cells (Li et al, ACS Nano 2014, 10280) and incubate with MoS2 nanosheets. The oxidation damages of lysosomal membrane were examined by lipid peroxidation kit. As shown in Figure R10, MoS2 induced significant oxidation to isolated lysosomes, whereas surface passivation could significantly ameliorate this oxidation damage. This result has been added in revised manuscript as Figure S10, and described in line 245 to 249, as “We used isolated lysosomes from BEAS-2B cells to examine the oxidation capability of MoS2 and me-MoS2 by the lipid peroxidation staining kit. As shown in Figure S10, MoS2 induced significant oxidation of isolated lysosomes emitting strong fluorescence of oxidized BODIPY 581/591 C11, whereas surface passivation could fully ameliorate this effect.” Besides, we have confirmed the lipid extraction capability of WS2 using liposome and red blood cell models (Figure 2d). We hope the

19

new data strengthened our speculations on the interactions between surface vacancies and lysosomal membranes.

25000

20000

15000

10000

**Fluorecence Intensity (a.u.)**

5000

0

30000

*

| **Ctrl MoS2** | **me-M oS2** |
| --- | --- |

**Figure R10 Oxidative damages of lysosomes by MoS2.**

Lysosomes extracted from BEAS-2B cells were incubated with 200 μg/mL MoS2 for 2 h. After centrifugation at 16000 rpm for 10 min, the supernatants were incubated with lipid peroxidation assay kit for 2 h to examine the oxidation damages in MoS2 treated lysosomes. *p< 0.05 compared to Ctrl by two-tailored Student T-test.

4.14 The author based a great deal of emphasis on the lysosomes and they presumed that these organelles are the source of iron in this setting, but they need to provide real evidence that the TMDs are in fact present in lysosomes, by specific labeling of these organelles. Showing TEM images of the TMDs in cells in not sufficient evidence. **Response:** Thanks for the reviewer’s suggestion. We used a flurophore to label WS2/MoS2 according to our previous reported method (Li et al, ACS Nano, 2018, 2, 1390-1402). Nuclei and lysosomes were labeled by Hoechst 33342 and lysotracker, respectively. Consistent with our TEM observation, the TMDs had a nice overlap with lysosomes (Figure R11). This result has been added in revised manuscript as Figure S4.

20

**Confocal visualizing TMDs in BEAS-2B cells**

**Nuclei	Lysosomes	TMDs	Overlay**

**Figure R11 Confocal imaging of cellular distribution of WS2 and MoS2**

WS2

Ctrl

MoS_2_

WS2 and MoS2 were labeled by FITC-BSA (green). BEAS-2B cells treated by 100 jig/mL WS2 and MoS2 for 24 h were stained by Hoechst 33342 (blue) and lysosomal tracker (red) to visualize the nuclei and lysosomes in cells, respectively (scale bar, 10 jim).

4.15 As a general remark, it is not clear why the authors have placed all the results with THP-1 cells in the supplement. These results should also be presented in the main text.

**Response:** Thanks for the reviewer’s comments. We have put some of the THP-1 cell data in main text, including cytotoxicity assessment in wide type (Figure 1C), knock down (Figure 3C) and Fer-1 treated cells (Figure 4A).

4.16 The authors have used knockdown of the transferrin receptor (TfR) to "validate" the role of iron. The method for this "knockdown" is not clarified in the manuscript: which cells were used?

**Response:** Thanks for the reviewer’s comments. We have added more details of the knockdown cell experiments in method sections (line 431-445), as “PLKO (24 jig, 14 jiL), shRNA (14.5 jig Trfc1, 13.3jig Trfc2, 14 jiL), SPA (17.9 jig, 14.5 jiL), pMD (6.0 jig, 5.3 jiL) and CaCl2 (2M, 187.5 jiL) solutions were sequentially added into a 2 mL Eppendorf tube with 1.5 mL DI H2O as DNA components. Aliquot of 1.5 mL N,N-bis(2-hydroxyethyl)-2-aminoethanesulfonic acid (BES) buffer was added into a

21

15 mL tube. Meanwhile, CaCl2 solution and DNA components were uniformly mixed with BES buffer and incubated for 15 min at room temperature. The resulting solution was added into 293T cells cultured till 50% confluency in Dulbecco's modified eagle medium (DMEM) medium (Gibco, USA) in a 75 cm^2^ flask. The post-transfection viral supernatants were collected twice at the 24 h and 48 h. Then the viral solution was acquired by centrifugation of collected supernatants at 3000 g for 15 min through a filter (0.45 μm pore size) and concentrated to 2 mL. Ultimately, aliquots of 20 μL viral solution were added into 10 mL BEGM or c-RPMI 1640 media to incubate with BEAS-2B and THP-1 cells with 50% confluency at 37 °C, respectively. After 24 h, the supernatants were replaced with complete media. The stable clones with GFP green fluorescence were selected in puromycin (10 μg/ml).”

4.17 The methods section mentions "shRNA transfectants of target genes (Trfc1, Trfc2)" (page 15) but which cells were used and where is the method description on the transfection of cells?

**Response:** Thanks for the reviewer’s comments. As we responded above, the details for cell transfection have been added in line 431-445 of revised manuscript.

4.18 Adding to this, the schematic figure (Figure 4) is completely redundant and it should be removed from the paper. This is a typical "graphical abstract" figure and does not belong in the main text (and it is unusual to "summarize" the findings in the middle of a paper. Furthermore, the figure does not show some of the key pieces of information, such as: the role of iron? (and the role, if any, of the transferrin receptor). As it stands, the figure only suggests that lysosomes and ROS are involved in so way in lipid peroxidation and cell death.

**Response:** Thanks for the reviewer’s suggestion. We have removed Figure 4 in revised manuscript.

4.19 The "mechanism" of lysosomal damage (i.e. phospolipid extraction and/or oxidation) is also very vague and this is, in fact, a major deficiency in the current study. The authors need to present more evidence for one or both of these potential mechanisms to strengthen the story.

**Response:** Thanks. As the reviewer suggested, we used a lysosomal isolation kit to acquire lysosomes from BEAS-2B cells (Li et al, ACS Nano 2014, 10280) and incubate with MoS2 nanosheets. The oxidation damages of lysosomal membrane were examined by lipid peroxidation kit. As shown in Figure R10, MoS2 induced significant oxidation to isolated lysosomes, whereas surface passivation could significantly ameliorate this oxidation damage. This result has been added in revised

22

manuscript as Figure S10. A description of this result has been added in line 245 to 249, as “We used isolated lysosomes from BEAS-2B cells to examine the oxidation capability of MoS2 and me-MoS2 by the lipid peroxidation staining kit. As shown in Figure S10, MoS2 induced significant oxidation of isolated lysosomes emitting strong fluorescence of oxidized BODIPY 581/591 C11, whereas surface passivation could fully ameliorate this effect.”

4.20 They also need to provide more evidence that the passivation strategy actually has an impact on these mechanisms of lysosomal damage as opposed to a more general effect. For instance, surface passivation may simply lead to a reduced cellular uptake of the TMDs thereby leading to a reduced toxicity. In other words, the passivation does not necessarily have to mping on "ferroptosis" signaling in cells.

**Response:** Thanks for the reviewer’s comments. To testify the possibility that passivation may simply lead to a reduced cellular uptake of TMDs, we examined the cellular uptake of pristine and modified WS2 and MoS2 nanosheets. As shown in Figure R12, surface passivation did not significantly change the cellular internalization of WS2 or MoS2 nanosheets. This result has been added in revised manuscript (Figure S11), and described in line 249 to 250, as “Biocompatibility test in cells showed that the modified nanosheets had similar cellular uptake to pristine TMDs (Figure S11)”

**WS2	MoS2**

PristineTMDs Modified TMDs

40

35

30

25

20

15

10

5

0

**Metal ion content (*μ*g/mg protein)**

50

45

**Figure R12 Impacts of surface passivation on cellular uptake**

BEAS-2B cells were exposed to WS2, MoS2, ss-WS2 and me-MoS2 at the concentration of 100 μg/mL for 24 h. Then the cells were collected, sufficiently washed and digested for ICP-OES detection.

23

4.21 It is also noted that the experiment using TfR "knockdown" cells were conducted at a dose of 200 ug/mL (48 hours): this is very excessive and the authors need to address the dose-response, if any.

**Response:** Thanks for the reviewer’s comments. We have tested the dose-response effects of WS2 and MoS2 in KD cells. As shown in Figure R13, while WS2 had no effects to the viability of KD cells, MoS2 showed a little toxicity at the highest dose.

**MTS@48h**

120

**TfR-KD BEAS-2B cell viability (%)**

100

80

60

40

20

0

WS2 MoS2

0 50 100 150 200
**Concentration (μg/mL)**

**Figure R13 Cell viability test in TfR knockdown cells**

TfR-KD BEAS-2B cells were exposed to WS2 and MoS2 at 0-200 μg/mL for 48 h.

The cell viability was examined by MTS assay.

4.22 As already mentioned, the in vivo part is unsatisfactory and the results do not "duplicate the results seen in tissue culture cells". The animal study shows that the TMDs elicit pulmonary inflammation with increased expression of a couple cytokines.

**Response:** As we responded to comment 4.9, we have added more results to validate ferroptotic cell deaths by examine Fe^2+^ release, lipid peroxidation and GPX-4 expression in animal lungs (Figure 6). We also observed the protection effects of Fer-1 on WS2 induced lung toxicity. We hope these new data strengthen our claim that TMD-induced ferroptosis could be validated in animal lungs.

4.23 The authors also stated that macrophage numbers were significantly increased in BAL fluid" but they do NOT show these data (Figure 6A shows images of iron labeling in alveolar macrophages). Instead, what they show is that neutrophil numbers are increased (Figure 6B).

**Response:** Thanks for pointing out this mistake. We actually mean neutrophil numbers, rather than macrophage numbers. We have corrected this in revised

24

manuscript.

4.24 Why did the authors not address whether there is any ferroptotic cell death in the lungs of mice? (and whether Trolox or other antioxidants prevents the cell death). **Response:** Thanks for the comments. We have addressed these concerns in our responses to comment 4.8 and 4.9.

1200

4.25 Conversely, do TMDs elicit any cytokine release in THP-1 or BEAS-2B cells? (in particular, IL-1beta or TNF-alpha).

1000

**Response:** To accommodate the reviewer’s concern, THP-1 cells were exposed to TMDs and monosodium urate (positive control) for 24 h. IL-1β was detected in

80

supernatants by ELISA. As show in Figure R14, only WS2 at 200 μg/mL elicited significant IL-1β production, whereas other TMDs had negligible effects. We think this is a very interesting phenomenon deserving more exploration in our future work.

6 0

**Figure R14 IL-1β production in TMD-treated THP-1 cells**

*

500

200 μg/mL

50 μg/mL

**IL-1 β (pg/mL)**

400

300

200

100

0

THP-1 cells were exposed to 50 or 200 μg/mL MSU, WS2, MoS2, WSe2 and MoSe2 for 24 h. The supernatants were collected to determine IL-1β production by ELISA (n=3). *p<0.05 compared to ctrl by two-tailored Student T-test.

5. Discussion

5.1 Unfortunately, and similarly to the introduction, this section is unsatisfactory, and very brief. The authors do not provide a sufficient discussion (here or in the introduction) of other previous studies of MoS2 or other 2D materials even though some previous studies have shown very similar results (for instance, Wang X et al. 2015;11(38):5079-5087).

**Response:** Thanks for the comments. To accommodate the reviewer’s concern, we have added more discussions on the contrary reports of TMD toxicities in line 320 to 333, “The differences of material source, physicochemical properties of TMDs, exposure time, doses and routes may be responsible for the conflict reports. Wang *et*

25

*al* used same cell lines (THP-1 and BEAS-2B) to us for cytotoxicity assessments and found limited cell viability changes at 24 h incubation with 0-50 μg/mL MoS2 nanosheets, whereas we merely observed significant cytotoxicity at 48 h incubation with 50-200 μg/mL MoS2 nanosheets, indicating that the exposure time and doses of TMDs may greatly affect their cytotoxicity. In terms of the impacts of exposure routes, Mei et al’ study showed that intravenous injected MoS2 elicited more toxicity than intraperitoneal and intragastric administration^11^. Under similar doses, same exposure time and route, we and Wang *et al* discovered similar pulmonary inflammation effects for MoS2 nanosheets. Besides, consistent with our nano-SAR findings, the surface chemistry of TMDs may play a major role in their toxicities, evidenced by the increased biocompatibility of MoS2 nanosheets functionalized by Pluronic 127^42^, Pluronic 87^9^ and PEG molecules^43^.”

5.2 The authors, again, exaggerated the conclusion regarding "nano-SARs". One cannot claim that the present study can "determine the tiered hazard signaling pathways and nano-SARs of TMDs in mammalian cells". The present study has NOT elucicated any hazard tiers and cannot be generalized to all TMDs nor to all mammalian cells. The authors MUST focus simply on the results at hand.

**Response:** Thanks for the reviewer’s comments. As responded to comment 1.1 and 2.3, we have added more data to support our claims on nano-SARs. We also revised our conclusion to avoid exaggeration. We hope the conclusion section is significantly improved in revised manuscript.

5.3 The authors also claim (on page 14) that "as expected, MoS2 and WS2 induced ferroptosis in alvealar macrophages and acute inflammation in lung tissues" but this is not correct. They did NOT show ferroptosis (or any other form of cell death) in the animal model (and the results cannot be considered to be "expected"). More work is required to link the in vitro and in vivo findings.

**Response:** Thanks for the comments. As responded to comment 4.9, we have added more data to validate ferroptosis in animal lungs. We hope the new results significantly strengthen the link between *in vitro* and *in vivo* findings.

5.4 The authors did not consider the differences in possible bio-coronas under in vitro and in vivo conditions but this may also be very relevant especially if the TMDs cause oxidation of PC (a major component of the lung surfactant fluid).

**Response:** Thanks for the comment. We agree with the reviewer that the formation of lipid corona on TMDs may be also relevant to their toxicity. It’s high likely that TMDs may cause oxidation of the surfactants in animal lungs and elicit lung toxicities.

26

This is a very interesting hypothesis and we will explore more in our future study. A short discussion about this has been added in line 306 to 312 of revised manuscript, as “Beside of the direct impacts of surface vacancy on cell viability, inhaled TMD nanosheets may escape the clearance by mucociliary escalator, deposit in pulmonary alveoli and interact with lung surfactants and surfactant proteins to form bio-corona structures^40^. It’s highly possible that the surface vacancy on MoS2 may elicit the oxidation of unsaturated lipids. These oxidized lipids are able to bind with the ΔN59 domain of caspase 11 for activation of inflammasomes^41^, which may further lead to pulmonary inflammation.”

6. Figures: The figure legend of Figure 6 ("Validation of ferroptosis and nano-SAR in

mouse lungs") is entirely misleading.

**Response:** Thanks. We have revised the figure legend.

**Reviewer comments, second version:**

Reviewer #1 (Remarks to the Author):

Since the authors clarified most of my questions, I agree with reviewer 2 points.


Reviewer #2 (Remarks to the Author):

I'm satisfied with changes and manuscript can be published.


Reviewer #3 (Remarks to the Author):

Xu et al. have significantly revised the manuscript and they added new data to support their claims. In partcular, they have now synthesizd and screened a lage number of TMDs (Table 1) and the results seem to support the idea of a so-called nano-SAR. The authors also performed experiments using ferrostatin-1 to provide more evidence for a lipid peroxidation-dependent cell death and they have expanded on the in vivo validation studies.

Having said that, there are some remaining issues and questions:

1. Table 1: The legend of this table does not describe how the cell viabliity (%) results were obtained: which cell line, what dose, what duration of exposure, and which cell viability assay?

2. The authors claim that ferrostatin-1 is a "specific" ferroptosis inhbitor but this is not correct; ferrostatin-1 is a radical-traping antioxidant (ACS Cent Sci. 2017 Mar 22;3(3):232-243). The inhibition of cell death by ferrostatin-1, combined with the fact that zVAD-fnk fails to prevent cell death, supports the notion that cell death is through ferroptosis (a non-apoptotic cell death). The effect of Trolox also supports this conclusion. The authors also stated several times that the have notd "Fe2+ release" but this statement (see abstract, and main text) is ambiguous and the authors have only mentioned in a few places in the text that they are referring to lysosomal release of Fe2+ into the cytoplasm (as opposed to, for instance, cellular release of Fe2+ into the extracellular space). They should clarify that they mean "lysosomal release of Fe2+". The authors have also used a cell model with knockdown of the transferrin receptor, and they showed that there is less cell death in these cells, but what is the relevance of knocking down the transferrin receptor (a receptor that brings transferrin into the cell) if the iron that is required for ferroptosis is released from lysosomes? The authors have not explained the role of extracellular versus intracellular iron in the present study and the experiments using TfR knockdown cells seem out of place.

3. There are some other minor issues: The authors now speculate that MoS2 may elicit the oxidation of unsaturated lipids (Discussion) but this does not actually address the previous comment which was: what is the role of the bio-corona in vitro and in vivo and do TMDs cause oxidation of lung surfactant? Moreover, the statement regarding the possible binding of oxidized lipids to the N59 domain of caspase-11 for activation of inflammasomes is entirely unsubstantiated and this should be removed as the present study does not touch at all on inflammasome activation (incidentally, inflammasome activation is caspase-dependent and the present study suggests a caspase-independent action of TMDs so this speculation is of little value).

4. Very minor: the last sentence of the Discussion should probably read "safe design of TMD nanoproducts such as lubricants" (add "such"). "Parkinsonian syndrome" (abstract) should read "Parkinson's disease". The statement in the Introduction concerning "arouse massive research enthusiasms on" shuld be read "arouse great enthusiasm for" or "stimulate considerable attention among researchers for". The "nice colocalization" with lysosomes (Results) should simply read "colocalization". References: the title is missing for ref. 40. Figure legends: the "Hazard signals in TMD-induced ferroptosis" should read "Biomarkers of ferroptosis in TMD-exposd cells".

**Author rebuttal, second version:**

**Reviewer: 1**

Comments:

Since the authors clarified most of my questions, I agree with reviewer 2 points.

**Response:** It’s glad to hear our responses clarified most of the referee’s questions.

**Reviewer: 2**

Comments:

I'm satisfied with changes and manuscript can be published.

**Response:** Thanks for agreeing that our manuscript can be published.

**Reviewer: 3**

Comments:

Xu et al. have significantly revised the manuscript and they added new data to support their claims. In partcular, they have now synthesizd and screened a lage number of TMDs (Table 1) and the results seem to support the idea of a so-called nano-SAR. The authors also performed experiments using ferrostatin-1 to provide more evidence for a lipid peroxidation-dependent cell death and they have expanded on the in vivo validation studies.

**Response:** Thanks for thinking that our new data supported the claims.

Having said that, there are some remaining issues and questions:

1. Table 1: The legend of this table does not describe how the cell viabliity (%) results were obtained: which cell line, what dose, what duration of exposure, and which cell viability assay?

Response: Thanks for the suggestions. We have added the descriptions of cell viability tests in the legend of Table 1, as “Cell viability data were acquired in BEAS-2B cells exposed to 200 μg/mL for 48 h by MTS assay.”

2. The authors claim that ferrostatin-1 is a "specific" ferroptosis inhbitor but this is not correct; ferrostatin-1 is a radical-traping antioxidant (ACS Cent Sci. 2017 Mar 22;3(3):232-243). The inhibition of cell death by ferrostatin-1, combined with the fact that zVAD-fnk fails to prevent cell death, supports the notion that cell death is through ferroptosis (a non-apoptotic cell death). The effect of Trolox also supports this conclusion. The authors also stated several times that the have notd "Fe2+ release" but this statement (see abstract, and main text) is ambiguous and the authors have only mentioned in a few places in the text that they are referring to lysosomal release of Fe2+ into the cytoplasm (as opposed to, for instance, cellular release of Fe2+ into the extracellular space). They should clarify that they mean "lysosomal release of Fe2+". The authors have also used a cell model with knockdown of the transferrin receptor, and they showed that there is less cell death in these cells, but what is the relevance of knocking down the transferrin receptor (a receptor that brings

transferrin into the cell) if the iron that is required for ferroptosis is released from lysosomes? The authors have not explained the role of extracellular versus intracellular iron in the present study and the experiments using TfR knockdown cells seem out of place.

**Response:** Thanks for these comments. To accommodate the reviewer’s concern on ferrostatin-1, we deleted “specific” in revised manuscript. We also agree with the reviewer that “lysosomal release of Fe^2+^” is a more accurate description. We have used this phrase to replace “Fe^2+^ release” in revised manuscript.

Transferrin receptor is very relevant to ferroptosis because i) it has been identified as a specific ferroptosis marker (Feng *et al*, Cell Reports, 2020, 3411-3423); ii) knockdown of TfR could affect the uptake of extracellular iron and reduce iron storage in lysosomes as well as the lysosomal release of Fe^2+^ (Elizabeth *et al*, Antioxidants & Redox Signaling, 2010, 997-1030). Therefore, TfR knockdown is a reliable method to examine ferroptotic cell death, evidenced by improved cell viability (Gao *et al*, Molecular Cell, 2015, 298-308; Ma *et al*, Cell Death Dis, 2016, e230). Consistently, TfR knockdown significantly ameliorate TMD-induced cell deaths, supporting our claim on TMD-induced ferroptosis. To accommodate the reviewer’s concern, we have added a short clarification of the relevance of TfR knockdown in line 164, as “Since transferrin receptors (TfR) serving as the dominant transporter of iron in cell metabolism is very relevant to ferroptosis^27^, TfR knockdown (TfR-KD) is a reliable method to examine ferroptotic cell death28, 29”

3. There are some other minor issues: The authors now speculate that MoS2 may elicit the oxidation of unsaturated lipids (Discussion) but this does not actually address the previous comment which was: what is the role of the bio-corona in vitro and in vivo and do TMDs cause oxidation of lung surfactant? Moreover, the statement regarding the possible binding of oxidized lipids to the N59 domain of caspase-11 for activation of inflammasomes is entirely unsubstantiated and this should be removed as the present study does not touch at all on inflammasome activation (incidentally, inflammasome activation is caspase-dependent and the present study suggests a caspase-independent action of TMDs so this speculation is of little value).

**Response:** Thanks for the suggestions. We have removed the discussion on the binding of oxidized lipids to the N59 domain of caspase-11. To address the reviewer’s concern on the role of bio-corona *in vitro* and *in vivo*, we have added a short discussion in line 318 to 323, as “The adsorption of immunoglobulins, complement factors, lipids and coagulation proteins on TMD surfaces may lead to the recognition and capture by immune cells *in vivo*, eliciting rapid clearance and significant immunotoxicity44, ^45^. In contrast, formation of protein corona in vitro may reduce the cellular internalization of nanoparticles and ameliorate cytotoxicity due to improved biocompatibility43, 46”

4. Very minor: the last sentence of the Discussion should probably read "safe design of TMD nanoproducts such as lubricants" (add "such"). "Parkinsonian syndrome" (abstract) should read "Parkinson's disease". The statement in the Introduction concerning "arouse massive research enthusiasms on" shuld be read "arouse great enthusiasm for" or "stimulate considerable attention among researchers for". The "nice colocalization" with lysosomes (Results) should simply read "colocalization". References: the title is missing for ref. 40. Figure legends: the "Hazard signals in TMD-induced ferroptosis" should read "Biomarkers of ferroptosis in TMD-exposd cells".

**Response:** Thanks for the suggestions. We have made corrections in revised manuscript.

**Reviewer comments, third version:**

**Author rebuttal, third version:**
